# Annual Research Review: Psychosis in children and adolescents: key updates from the past 2 decades on psychotic disorders, psychotic experiences, and psychosis risk

**DOI:** 10.1111/jcpp.14088

**Published:** 2025-01-03

**Authors:** Ian Kelleher

**Affiliations:** ^1^ Division of Psychiatry, Centre for Clinical Brain Sciences University of Edinburgh Edinburgh UK; ^2^ School of Medicine University College Dublin Dublin Ireland; ^3^ School of Medicine University of Oulu Oulu Finland; ^4^ St. John of God Hospitaller Services Group Hospitaller House, Stillorgan Dublin Ireland

**Keywords:** Schizophrenia, early‐onset psychosis, psychotic experiences, psychosis risk, children, adolescents

## Abstract

Psychosis in children and adolescents has been studied on a spectrum from (common) psychotic experiences to (rare) early‐onset schizophrenia spectrum disorders. This research review looks at the state‐of‐the‐art for research across the psychosis spectrum, from evidence on psychotic experiences in community and clinical samples of children and adolescents to findings from psychosis risk syndrome research, to evidence on early‐onset psychotic disorders. The review also looks at new opportunities to capture psychosis risk in childhood and adolescence, including opportunities for early intervention, identifies important unanswered questions, and points to future directions for prevention research.

## Introduction

Psychosis in children and adolescents has been studied on a spectrum from (common) psychotic experiences to (rare) early‐onset schizophrenia spectrum disorders. This annual research review starts with an exploration of research on psychotic experiences in children and adolescents, including information on their prevalence, clinical significance, typical course, and the role of interventions in young people with psychotic experiences. Next, it looks at ‘high risk’ approaches to the prediction of psychotic disorders, including clinical and familial high‐risk paradigms, and how they specifically apply to children and adolescents, as well as looking at recent research on psychosis risk in general child and adolescent psychiatry services and other services or systems. It then looks at research on early‐onset psychosis, including research on risk factors, pharmacotherapy and psychosocial interventions, as well as highlighting areas for further research.

## Psychotic experiences in children and adolescents

There has been intense research interest in psychotic experiences over the past 25 years, also termed ‘psychotic‐like experiences’ and ‘psychosis spectrum symptoms’ (Crush et al., 2018; Ellersgaard et al., 2021; Healy et al., 2019; Johns & Van Os, 2001; Kelleher et al., 2012; McGrath et al., 2015; Poulton et al., 2000; Rimvall et al., 2020; Scott, Chant, Andrews, & McGrath, 2006; Trotta et al., 2020). This research has focussed largely on positive psychotic phenomena in the general population, typically outside of the context of psychotic disorders, especially unusual perceptual experiences, such as hearing voices or sounds that others do not hear (Bartels‐Velthuis, van de Willige, Jenner, van Os, & Wiersma, 2011; Maijer et al., 2019; Scott et al., 2009).

**Figure 1:**
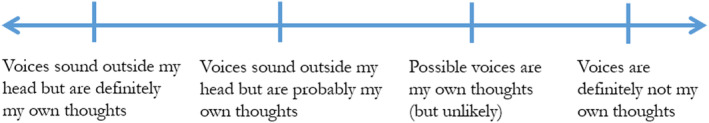
Insight in the context of psychotic experiences. In this example, unusual auditory experiences (hearing voices) may be mapped somewhere from occurring in the context of full insight (on the left) to frank psychosis (on the right). Most psychotic experiences do not meet the criteria for frank psychosis

Psychotic experiences typically differ from full‐threshold psychotic symptoms in that most young people who report psychotic experiences have at least some degree of insight, which is lacking in frank psychosis. For example, a young person may perceive voices when no one is speaking but know that these experiences are arising from their own mind. The level of insight can be mapped on a continuum, see Figure 1.

New or incident psychotic experiences are reported by about two in 100 individuals per year but they are substantially more common in younger populations: incident psychotic experiences are reported by approximately five in 100 adolescents per year (Staines et al., 2023).

There has been less research on psychotic experiences in clinical (as opposed to community) samples but a number of studies have demonstrated that these phenomena are very common in clinical populations. Studies from Canada, Ireland, and the Netherlands have shown that up to half of teenagers attending specialist mental health services report psychotic experiences (Cleverley et al., 2023; de Jong et al., 2018; Kelleher et al., 2014).

Much of the initial interest in childhood psychotic experiences stemmed from the Dunedin study finding that they were associated with an increased risk of subsequent schizophreniform disorders when followed into adult life (Poulton et al., 2000). Psychotic experiences, then, were frequently conceptualised as a valuable approach to understanding early risk for psychotic disorders.

Whilst several longitudinal studies [though not all, e.g. (Dhossche, Ferdinand, van der Ende, Hofstra, & Verhulst, 2002)] have replicated the finding of an increased risk of schizophrenia spectrum disorders in young people who report psychotic experiences (Dominguez, Wichers, Lieb, Wittchen, & van Os, 2011; Fisher et al., 2013; Welham et al., 2009; Zammit et al., 2013), other research has painted a more complex picture on the clinical significance of psychotic experiences. Both community and clinical studies have demonstrated that psychotic experiences are not uniquely related to any particular diagnostic group; rather, they are prevalent across all child and adolescent mental disorder diagnoses, including affective, anxiety, neurodevelopmental, behavioural, and substance use disorders [see Healy et al. (2019) for review]. There is a more limited number of longitudinal studies investigating adult outcomes of childhood/adolescent psychotic experiences but the existing literature has shown an increased adult incidence of depressive disorders (Dhossche et al., 2002), post‐traumatic stress disorder (Fisher et al., 2013), substance use disorders (Cederlöf et al., 2017; Dhossche et al., 2002) and suicidal behaviour (Cederlöf et al., 2017; Dhossche et al., 2002; Fisher et al., 2013).

It is notable that psychotic experiences are not always associated with psychopathology, especially when they occur in younger children. Research suggests, however, that psychotic experiences become more strongly associated with psychopathology from the teenage years onward. General population research cutting across childhood and adolescence, for example, has shown that psychotic experiences are associated with diagnosable psychopathology in approximately half of cases in pre‐teens, rising to approximately 80% in teenagers (Kelleher et al., 2012).

Research has also suggested that psychotic experiences are markers of risk for more severe psychopathology. For example, compared with individuals with a diagnosable mental disorder who do not have psychotic experiences, young people with a diagnosable mental disorder and co‐occurring psychotic experiences have more mental health service use (Bhavsar et al., 2017; Rimvall et al., 2020), greater multimorbidity (Kelleher, Keeley, et al., 2012) and poorer socio‐occupational functioning (Wigman et al., 2014).

Several studies in adults have demonstrated an association between psychotic experiences and poorer responses to both pharmacological (Perlis et al., 2011) and psychological (Knight et al., 2020; Wigman et al., 2014) treatments. However, these findings have not been replicated in studies of children and adolescents (Davies et al., 2020; Rimvall et al., 2024). In the Mind My Mind trial, for example, researchers did not find that the effectiveness of CBT in 6‐ to 16‐year‐olds was modified by the co‐occurrence of psychotic experiences (Rimvall et al., 2024).

One of the most consistently replicated findings on psychotic experiences has been a strong relationship between self‐harm and suicidal behaviour (DeVylder, Lukens, Link, & Lieberman, 2015; Hielscher et al., 2020; Kelleher et al., 2013; Nishida et al., 2008; O'Hare, Poulton, & Linscott, 2021; Saha et al., 2011). A systematic review of longitudinal studies found that psychotic experiences are particularly strongly associated with more severe suicidality, including a threefold increased odds of suicide attempt and a fourfold increased odds of suicide death (O'Hare et al., 2024). This may be in part due to the fact that they index risk for more severe mental illness but studies have found that psychotic experiences predict future suicidal behaviour in excess of that predicted by information on co‐occurring psychopathology alone (DeVylder et al., 2015; Hielscher et al., 2021; Narita, Wilcox, & DeVylder, 2020; Yates et al., 2019).

### Course of psychotic experiences in children and adolescents

A systematic review found that ~31% to 36% of children and adolescents who reported psychotic experiences had one or more additional psychotic experiences within a year (Staines, Healy, Murphy, et al., 2023). Persistent psychotic experiences were associated with an increased risk of mood disorders, psychotic disorders, substance abuse disorders, suicidal ideation, and suicidal behaviour (Staines, Healy, Murphy, et al., 2023). Trauma, anxiety, and high overall psychopathology scores were found to be associated with persistent psychotic experiences. One study aimed to look at the effect of substance use on psychotic experience persistence in children and adolescents but the numbers with any substance use (*n* = 3) were too low to draw any conclusions (Bartels‐Velthuis et al., 2011).

### Intervention studies for psychotic experiences

There has been limited research to date on the treatment of psychotic experiences. Their high prevalence across the full spectrum of mental disorders, however, points to the importance of identifying and treating underlying or co‐occurring mental health disorders, be they psychotic disorders (uncommon) or non‐psychotic disorders (common).

A number of studies have looked at the secondary effects of (non‐psychosis specific) interventions on psychotic experiences. One randomised controlled trial (RCT) investigated the effect of cognitive behavioural therapy for insomnia (CBTi) on sleep in university students and found that not only did it improve sleep but it also reduced psychotic experiences (Freeman et al., 2017).

Another trial carried out on college students found that a transdiagnostic ‘resilience training’ intervention was associated with reduced psychotic experiences at follow‐up compared with waitlist control. Of note, however, randomisation to waitlist control groups may be associated with poorer outcomes than simply receiving no psychological intervention (Patterson, Boyle, Kivlenieks, & Van Ameringen, 2016), meaning these results must be interpreted cautiously.

An RCT on the prevention of suicidal behaviour in adolescents (Saving and Empowering Young Lives in Europe; SEYLE) found that input from specialist mental health services (but not a universal school‐based intervention nor a teacher training intervention) was associated with a significantly reduced risk of psychotic experiences at follow‐up (Staines et al., 2023). Mental health service input was triggered by high psychopathology scores and self‐harm/suicidal behaviour as rated on a series of questionnaires, rather than relating to psychotic experiences per se. Whilst it was not possible to identify what aspects of mental health service input led to reduced psychotic experiences, overall, the results from existing trials point to the possibility that interventions aimed at problems associated with psychotic experiences (sleep problems and general mental health problems), rather than aimed at the psychotic experiences themselves, may have positive indirect effects on psychotic experiences. Further research is needed in this area.

Further research is also needed to clarify whether certain features of psychotic experiences are more important than others, for example, in terms of psychopathological significance, such as phenomenological aspects of the experiences (McCarthy‐Jones, Krueger, Larøi, Broome, & Fernyhough, 2013; Schutte et al., 2020), their frequency (Shevlin, Boyda, Houston, & Murphy, 2015; Wüsten et al., 2018), timing (DeVylder & Kelleher, 2016), emotional valence (Underwood, Peters, & Kumari, 2015), or associated distress (Bak et al., 2005; Murphy, McBride, Fried, & Shevlin, 2018; Oh et al., 2024).

**What do I tell parents and young people about psychotic experiences? Pointers for clinicians**

- *Contextualise*: Unusual perceptual experiences are more common than most people realise (up to one in five children report ‘hearing voices’ or similar phenomena). For the majority of young people, these experiences are transient.
- *Educate*: It can be helpful to explain that adolescence is a period of rapid brain development and reorganisation. Some connections between brain cells and regions are becoming stronger and reinforced whilst less important connections may be removed (pruned) to make the brain more efficient. These changes affect how different parts of the brain connect and communicate with each other and it is likely that whilst this reorganisation is happening, errors in brain communication (or ‘crossed wires’) are more likely to happen. This may make unusual perceptual experiences (such as a thought sounding as if it were spoken aloud) more likely. Unusual perceptual experiences are more likely to occur in young people with a broad range of mental health and neurodevelopmental conditions. They can also occur in people with no mental health/neurodevelopmental condition.
- *Reassure*: Some parents will worry about the risk of psychotic disorder. Reassure that unusual perceptual experiences are not inherently an indicator of emerging schizophrenia/psychotic disorder. Only a small proportion of young people (less than one in 10) who report unusual perceptions go on to develop a psychotic disorder, which means that >90% do not. Where unusual perceptual experiences persist or are very distressing, parents should be advised to discuss this with their doctor.
- *Treat (where indicated)*: There is limited evidence on interventions for psychotic experiences. It is important to remember that unusual perceptual experiences are not inherently pathological and may emerge even in the context of normal brain development – so an intervention is not automatically indicated. The focus should typically be on identifying if there are underlying mental health problems (which may be contributing to the emergence or persistence of psychotic experiences) and addressing these. For example, if the young person has a specific mental health disorder, treating this may also lead to reduced psychotic experiences. Similarly, addressing any underlying stressors may lead to an improvement. General principles of good mental health are also likely to apply here, including advice around good sleep, exercise, structure, and avoiding substance use.

## High‐risk approaches in children and adolescents

### Clinical high risk for psychosis

A related research stream to the study of psychotic experiences in the population has been the development of the clinical high‐risk (CHR) approach to psychosis prediction (also called the ultra high risk and at risk mental state approach). The CHR approach involves using structured interviews to assess attenuated psychotic symptoms (or brief, full‐threshold psychotic symptoms) in people typically aged between 12 and 35 years old (Addington & Heinssen, 2012; Yung, Phillips, Yuen, & McGorry, 2004). Depending on the frequency and severity of symptoms, someone may be diagnosed as having a CHR state. Research (predominantly in young adults, rather than children and adolescents) suggests that ~25% of these individuals develop a psychotic disorder by 3‐year follow‐up (De Pablo et al., 2021), though there is wide variation in transition rates reported between studies.

The CHR approach has had a big impact on clinical services in many countries, as well as impacting on the Diagnostic and Statistical Manual of Mental Disorders (DSM) 5, with the addition of the ‘Attenuated Psychosis Syndrome’ (which largely reflects a CHR diagnosis) as a condition for further study (Zachar, First, & Kendler, 2020). It is important to note, however that whilst CHR services are typically open to individuals from age 12 years old, the majority of participants in CHR studies have been adults aged 18 years+.

A systematic review of CHR studies specifically in children and adolescents (age <18 years) found a wide variation in transition rates to psychosis (Lång et al., 2022). Depending on the study in question, the 2‐year follow‐up risk of psychosis varied from 3% to 20% and the 5‐year follow risk varied from 7% to 34%. The overall pooled transition rate to psychosis was 12% at 2‐year follow‐up and 16% at ≥5‐year follow‐up. A systematic review of CHR studies that included individuals with a mean age of 18 (i.e. not all participants were aged <18) found a pooled transition rate to psychosis of 23% at 2 years and the same (23%) at 3 or more years (Catalan et al., 2021).

Whilst the above findings suggest an overall increased risk of psychosis in children and adolescents diagnosed with CHR syndromes, albeit with an imprecise point estimate, there is a lack of evidence in children and adolescents as to whether an increased risk of psychosis is related to the young person meeting CHR criteria or whether the risk is enriched by the recruitment strategies involved in CHR studies (i.e. recruiting young people who are typically help‐seeking for their mental health). Lång et al. (2022) identified four studies that included comparison groups that were recruited using the same strategy as the individuals diagnosed with a CHR state (Lindgren et al., 2014; Manninen et al., 2014; Pelizza et al., 2018; Spada et al., 2016). These studies did not demonstrate a significantly higher risk of psychosis in the CHR samples relative to their matched controls. Thus, it is unclear what, if anything, a CHR diagnosis adds in terms of risk, beyond selecting from risk‐enriched groups.

Notably, one Australian study involved a 10‐year follow‐up of all individuals (ages 12–25 years) referred to a CHR service and found that whilst 17% of referred patients who met CHR criteria went on to develop a psychotic disorder, so too did 15% of referred patients who had not met CHR criteria (Conrad et al., 2017). This suggests that referral pathways can automatically capture significant risk for psychosis.

For young people who meet CHR criteria but do not transition to psychosis, a systematic review found that remission occurred in just under half (48.7%), meaning that about half continued to meet CHR criteria (De Pablo et al., 2022). Many studies have demonstrated that young people who meet CHR criteria also meet criteria for (non‐psychotic) mental disorders (Addington et al., 2017; Hui et al., 2013; Lee et al., 2013; Michel, Ruhrmann, Schimmelmann, Klosterkötter, & Schultze‐Lutter, 2018), meaning that remission from CHR status does not necessarily equate with good mental health.

### Sensitivity of the clinical high‐risk approach

In addition to looking at the absolute risk of psychosis associated with a CHR diagnosis, recent research has explored how sensitive CHR services are to identifying psychosis risk in the population: that is, looking at the total number of psychosis cases diagnosed in the population over a given period, what proportion had been captured by CHR services. This is important information because it tells us the upper limit of the total proportion of psychosis cases in the population that could be prevented if we had an effective preventive intervention. Studies from the UK and Australia suggest that only a small minority (4%–14%) of all future psychosis cases are captured by real‐world CHR services (Ajnakina et al., 2017; Burke et al., 2022).

Looking specifically at the sensitivity of CHR services for children and adolescents, a secondary analysis of Ajnakina et al.'s data from a south London CHR service found that only about 0.5% of future psychosis cases were captured by CHR services for children and adolescents (age <18 years) (Lång, Yates, et al., 2022). This suggests that offering CHR services to child and adolescent mental health services does not represent an effective strategy for psychosis prediction.

### Intervention studies in the clinical high‐risk approach

A number of trials have been conducted in CHR patients aimed at reducing the transition risk to psychosis, including integrated psychological interventions, cognitive behavioural therapy, family therapy, D‐serine, omega polyunsaturated fatty acids, minocycline, and antipsychotic medication (McGlashan et al., 2006; McGorry et al., 2017; Morrison et al., 2012; Qurashi et al., 2024; Stain et al., 2016) [see Davies et al. (2018) for review]. To date, evidence does not support any specific intervention for the reduction of psychosis risk in children and adolescents at clinical high risk (Davies et al., 2018).

Some researchers have suggested that declining rates of transition to psychosis seen over time in CHR studies may indicate that CHR interventions are, in fact effective. However, research has shown that the transition rate to psychosis in CHR centres that provide comprehensive psychological interventions, including, for example, individual case management, family interventions and cognitive – behavioural therapy, does not differ from the transition rate in ‘naturalistic’ CHR studies (studies that do not provide any specific interventions) (Simon et al., 2011). This suggests that something other than CHR interventions (such as recruitment or risk enrichment strategies) is responsible for declining transition rates.

It is not clear to what extent the lack of effectiveness demonstrated in CHR clinical trials to date relates to the interventions themselves, rather than the stage of illness captured by the CHR approach. Most of the transitions to psychosis in CHR patients occur in the first 1–2 years after CHR diagnosis and research suggests that the neurobiological changes underlying psychosis may already be advanced at that stage (Gomes, Rincón‐Cortés, & Grace, 2016; McCutcheon, Marques, & Howes, 2020; Paus, Keshavan, & Giedd, 2008). Indeed, some researchers have argued that psychosis, as typically emerges in adulthood, often reflects a late stage of a far longer‐standing neurodevelopmental disorder of childhood and adolescence (Insel, 2010). It may be the case that interventions need to be delivered further upstream to reduce psychosis risk.

It may also be the case that clinical trials need to follow CHR patients for longer periods of time than the usual 12‐month or 24‐month follow‐up to detect differences in outcomes, as it may only be individuals at earlier stages of illness development (more distal from a psychotic disorder) who benefit from these preventive interventions. It may also be the case, however, that the CHR state is a relatively advanced stage on the trajectory to psychotic disorder and that prevention is simply less likely to be effective at this stage. Further research is necessary to test these hypotheses.

With regard to the prediction and prevention of psychosis in children and adolescents using the CHR approach, the European Psychiatric Association (EPA) has issued the following guidance: ‘The EPA considers that the current evidence for the psychosis predictive value of CHR criteria and for the efficacy of psychological and pharmacological interventions in children and young adolescents is not sufficient to justify primarily preventive interventions’ (Schmidt et al., 2015).

### Familial high risk for psychosis

A familial high risk (FHR) for psychosis approach has also received considerable research attention, albeit less so than the CHR approach (Goldstein, Buka, Seidman, & Tsuang, 2010; Johnstone, Lawrie, & Cosway, 2002; Shah, Tandon, & Keshavan, 2013). The FHR approach involves identifying individuals with an elevated risk for psychosis, based on having relatives (typically one or both parents) with a history of psychosis. A recent systematic review found that ~8% of young people who meet FHR criteria go on to be diagnosed with a psychotic disorder (Solmi et al., 2022). Psychotic experiences are also more prevalent in the offspring of individuals with schizophrenia. In a study of Danish children at familial risk of schizophrenia, Gregersen and colleagues found that 32% reported psychotic experiences at age 11 (though, notably, psychotic experiences were also relatively common in the control group: children without a family history of schizophrenia, at 18%) (Gregersen et al., 2022).

### Sensitivity of the familial high‐risk approach

Previous research looking at population–attributable fractions for schizophrenia suggests that only a minority of cases are attributable to familial risk (Mortensen et al., 1999; Mortensen, Pedersen, & Pedersen, 2010). A recent total population study in Finland investigated the sensitivity of the FHR approach to capture future psychotic disorders (Healy et al., 2024). Looking at all individuals born between 1987 and 1993 and followed to age 28/29 years, 7% of all psychosis diagnoses and 9% of all schizophrenia diagnoses in the population occurred in individuals for whom one or both parents had previously received a psychosis diagnosis. The proportion of future psychosis cases captured using the FHR approach varied, however, according to the cut‐off age at which FHR was determined. If looking specifically at childhood, taking age 18 as the cut‐off age to determine FHR in offspring, then the sensitivity of this approach was 6% for all future non‐affective psychosis diagnoses and 7% for all future schizophrenia diagnoses.

This study also took a transdiagnostic approach to familial risk and identified the proportion of all psychosis cases that emerged in the offspring of parents who had a history of inpatient psychiatric admission for any reason (i.e. not limited to parental psychotic disorders). Using that transdiagnostic approach, 29% of all cases of psychotic disorders and schizophrenia in the population occurred in the offspring of parents with a history of any inpatient psychiatric admission. Again, the proportion of future psychosis cases captured using this transdiagnostic approach varied according to the cut‐off age at which familial risk was determined. If childhood (age <18) was taken as a cut‐off for determining risk, the sensitivity of this approach was 23% for all future non‐affective psychosis diagnoses and 25% for all future schizophrenia diagnoses.

### Intervention studies in the familial high‐risk approach

There have been limited trial/intervention studies to date in the FHR population. A number of factors, however, suggest that the FHR approach lends itself well toward preventive intervention, including that (1) FHR criteria are readily identifiable (based on routine administrative healthcare data) and (2) risk can be identified whilst still in childhood, which may be a more opportune time to impact on neurobiological, as well as psychosocial, development, rather than the early adult stage typically associated with CHR diagnoses.

One study, VIA Family, has recently reported its findings on children ages 6–12 years old with a parent who had a history of schizophrenia spectrum, bipolar, or depressive disorder (Müller et al., 2024). This study was a pragmatic, rater‐blinded, superiority trial with interventions individually tailored to the perceived needs of the family but which typically involved options for psychoeducation, parental support, and early treatment for emerging child psychiatric symptoms. At 18‐month follow‐up, there were no significant differences in outcomes for children in the active intervention arm compared with treatment as usual across a wide range of clinical and functional measures. Given the young age of the cohort, longer term follow‐up will be necessary to investigate if the VIA family interventions had any effect against the development of severe mental illness, including psychotic disorders.

Overall, further intervention trials are needed in FHR populations. Further research is also needed to identify subgroups at higher risk within the FHR population.

### Psychosis risk in young people attending child and adolescent psychiatry services

One additional childhood group of interest in terms of capturing psychosis risk is young people who attend specialist child and adolescent psychiatry services (Kelleher, 2023). We recently investigated psychosis risk associated with attendance of psychiatry services in childhood and adolescence (up to age 18) (Lång et al., 2022). Using total population healthcare register data, we followed all individuals born in Finland in 1987 from childhood up to age 28–29 years.

In total, 13% of all child and adolescent psychiatry (CAP) patients went on to be diagnosed with a psychotic or bipolar disorder. What is more, of all psychotic and bipolar affective disorders diagnosed in the population by age 29, half occurred in individuals who had attended CAP services. This included 54% of the total population diagnoses of schizophrenia and 52% of the total population diagnoses of bipolar disorder.

These findings suggest that whilst psychotic disorders are uncommon in childhood, specialist mental health services for children automatically create important opportunities for psychosis prediction and, ultimately, prevention. It is important to note that the above findings relate to risk captured in specialist child and adolescent mental health services and should not be generalised to young people meeting criteria for mental health disorders in other settings, such as primary care or community samples. Further research is needed to identify whether there are subgroups within CAP services who are at highest risk.

Young people attending specialist adolescent psychiatry services frequently meet the criteria for clinical high‐risk syndromes, including one finding that about a quarter of adolescents admitted to an inpatient psychiatric hospital for non‐psychotic disorders met CHR criteria (Salazar de Pablo et al., 2020). In keeping with research on psychotic experiences in adolescents attending psychiatric services (Kelleher et al., 2014), the authors found that adolescents who met CHR criteria had more severe (non‐psychotic) psychopathology, multimorbidity, and poorer functioning (Salazar de Pablo et al., 2020). Importantly, however, research that has followed adolescents attending psychiatric services over time did not find that those meeting CHR criteria had an elevated risk of psychosis compared with those who did not meet CHR criteria (Lindgren et al., 2014; Lindgren, Kuvaja, Jokela, & Therman, 2023), which can be explained by the fact that (as above) attending adolescent psychiatry services, in and of itself, is associated with substantially increased risk for later psychosis (Lång, Ramsay, et al., 2022).

### Intervention studies in young people attending child and adolescent psychiatry services

There have been no intervention studies aimed at reducing psychosis risk within the general CAP patient population to date. This population, however, holds promise for intervention because, as with the FHR paradigm, (1) the increased risk is identifiable based on routine administrative healthcare data (i.e. data that captures attendance at CAP services) and (2) risk is identified, by definition, whilst still in childhood, which, as above, may be a more opportune time to impact on neurobiological and psychosocial development. An advantage over the FHR (and CHR) approach is that (3) CAP services appear to capture a far higher proportion of future psychosis cases than either of the traditional (CHR and FHR) high‐risk approaches.

One approach that might be hypothesised to reduce the risk of psychosis amongst patients of child and adolescent mental health services is the treatment of childhood psychopathology. Whilst the idea of ‘intervention as prevention’ is heuristically appealing, there is little empirical evidence to support the idea that mental health treatments in childhood prevent poor mental health outcomes in adulthood, including psychosis [see Roest, de Vries, Wienen, & de Jonge (2023) for review]. This is an important area for future research. Given links between substance use and risk for psychosis, interventions aimed at reducing substance use in patients of CAP services might, if this relationship is causal, hold promise for reducing psychosis risk. This will also be an important area for future research.

Besides the treatment of childhood mental disorders and substance use, there may be other promising avenues of research for interventions to reduce psychosis risk in child and adolescent mental health services. Inflammation, for example, is believed to play an important role in the development of psychosis (Howes & McCutcheon, 2017; Mongan, Ramesar, Föcking, Cannon, & Cotter, 2020; Upthegrove, 2022), including its role in synaptic pruning and reorganisation of the adolescent brain (Paolicelli et al., 2011; Sakai, 2020). In that context, anti‐inflammatory and immunomodulatory treatments in patients of CAP services will be an important area for future study.

### Psychosis risk in children placed in out‐of‐home care

An additional system that has recently been shown to capture substantial risk for psychosis, in particular early‐onset psychosis, is being placed in out‐of‐home care in childhood (Kääriälä et al., 2022). One study followed all individuals born in Finland in 1997 up to age 18. Of all psychotic and bipolar disorders diagnosed before age 18 in the total cohort, 45% occurred in individuals who had entered out‐of‐home care. This highlights out‐of‐home care as particularly important for capturing risk for early‐onset psychosis. At the same time, it is important to note that the absolute risk for a psychotic or bipolar disorder in this group, at 4%, was still low (albeit exponentially increased compared with the rest of the population).

It is unknown how sensitive out‐of‐home care in childhood is for psychosis risk extending into adulthood. This will require further research.

One factor that links placements in out‐of‐home care and psychosis is childhood adversity. Whether adversity or trauma plays a direct causal role in the development of psychosis is not clear (Morgan & Fisher, 2007; Scott, Ross, Dorahy, Read, & Schäfer, 2018). It is also the case, for example, that parents with severe mental illness are more likely to have their children taken into out‐of‐home care (Nevriana et al., 2024), and thus, this group also experiences genetic transmission of risk.

## Early‐onset psychosis

Early‐onset psychosis and early‐onset schizophrenia refer to the onset of disorder prior to age 18 years (Xu et al., 2020). Research suggests that about 12% of psychotic disorders and 8% of schizophrenia cases have onset prior to age 18 (Solmi et al., 2022). Of all cases of early‐onset psychosis, a large majority of these cases emerge in adolescence (ages 13–17 years inclusive), with about 10% of all early‐onset psychotic disorders arising prior to age 13 years (Lemmers‐Jansen, Krabbendam, & van der Ven, 2023), which is referred to as childhood‐onset psychosis.

Whilst only a minority of cases of psychosis emerge before age 18, it is common for childhood psychopathology to precede psychosis (Maibing et al., 2015), and as described above, for individuals with psychotic disorders to have previously attended child psychiatry services for other (non‐psychotic) mental health problems (Lång, Ramsay, et al., 2022).

Research on childhood psychopathology preceding psychosis has focussed on attention deficit hyperactivity disorder (ADHD) and autism, primarily because, like psychosis, these conditions are considered neurodevelopmental in nature (Chisholm, Lin, Abu‐Akel, & Wood, 2015; Dalsgaard et al., 2014; Kincaid, Doris, Shannon, & Mulholland, 2017; Moran et al., 2019). However, recent research has shown that coming to CAP services for any reason is associated with an increased risk of subsequent psychosis and, in fact, that diagnoses of autism and ADHD may be associated with a *lower* risk of subsequent psychosis than other childhood psychiatry diagnoses, such as depression, anxiety, conduct disorders, or obsessive‐compulsive disorders (Lång, Ramsay, et al., 2022). Further research is needed to identify the reasons for a potential difference in risk between childhood neurodevelopmental disorders and other childhood mental disorders.

### Neuroimaging differences in early‐onset psychosis

The literature on neurobiological differences in early‐onset psychosis is relatively sparse compared with adult psychosis but a systematic review of structural and functional brain abnormality studies in early‐onset schizophrenia reported a number of differences compared with healthy controls (Ioakeimidis, Haenschel, Yarrow, Kyriakopoulos, & Dima, 2020). Functional magnetic resonance imaging (fMRI) studies showed reduced activation of the right anterior cingulate cortex and the right temporoparietal junction during general cognition. They also found consistent hypoactivation in the left precuneus, the right inferior parietal lobule, and the right temporoparietal junction during working memory tasks. Voxel‐based morphometry studies, on the other hand, did not find consistent evidence of reduced grey matter. Whilst differences compared with healthy controls are interesting, additional research is needed to compare young people with early‐onset schizophrenia to young people presenting to mental health services for other mental disorders as well as comparisons with adult‐onset psychosis.

### Outcomes of early‐onset psychosis

Early‐onset psychosis frequently has a poor prognosis, even relative to psychosis diagnosed in adulthood. A systematic review of prognostic studies found that just 15% of individuals with early‐onset schizophrenia and 20% of individuals with other early‐onset psychotic disorders had a ‘good’ outcome, defined as a global assessment of functioning score >70 (de Pablo et al., 2024). About one‐quarter of early‐onset schizophrenia patients and one‐third of individuals diagnosed with other early‐onset psychotic disorders had a ‘moderate outcome’, defined as a global assessment of functioning score of 51–70. Clearly, much further work is necessary to improve functional outcomes.

### Risk factors for early‐onset psychosis

Most of the same risk factors for adult‐onset psychosis also apply to early‐onset psychosis (Karlsgodt, 2023). There is evidence, however, that prenatal and perinatal factors, for example, fetal hypoxia, low birth weight, and maternal infection, may play even stronger roles in early‐onset psychosis (Rosso et al., 2000; Verdoux et al., 1997) than in adult‐onset psychosis (Clarke, Harley, & Cannon, 2006). Many schizophrenia‐candidate genes are known to be regulated by hypoxia, supporting a potential causal role of early brain insult in the development of early‐onset psychosis (Schmidt‐Kastner, Van Os, Esquivel, Steinbusch, & Rutten, 2012).

A recent study directly compared risk factors for early‐onset psychosis versus adult‐onset psychosis in 278 patients aged between 7 and 35 years old (Baeza et al., 2021) found a stronger association between the following risk factors and early‐onset psychosis (compared with adult‐onset psychosis): obstetric complications, birth weight <2,500 g, a prior mental disorder, a previous diagnosis of attention deficit/hyperactivity disorder, and a premorbid IQ of <85.

Cannabis use is one of the most studied putative risk factors in psychosis and appears also to be related to early‐onset psychosis. Animal model research has demonstrated that THC exposure disrupts synaptic pruning and maturation of the prefrontal cortex (Rubino et al., 2015), a key adolescent brain maturational process that is strongly implicated in schizophrenia. An observational study of 90 early‐onset psychosis patients compared with healthy controls found that lifetime use of cannabis was associated with a threefold increased odds of early‐onset psychosis, with daily cannabis use being associated with a 43‐fold increased odds of early‐onset psychosis (Pardo et al., 2021). Cannabis use before age 15 may be particularly risky (Pardo et al., 2021).

It may be the case that the relationship is bidirectional, with cannabis playing a causal role in the onset of psychosis, especially in those at increased genetic risk, whilst also being used as a form of self‐medication to try to manage symptoms (Ganesh & D'Souza, 2022; Karlsgodt, 2023). Overall, however, research suggests that the growing use of cannabis (and the increasing potency of cannabis) may be playing an increasing role in the development of psychotic disorders (Hjorthøj, Posselt, & Nordentoft, 2021).

### Treatment of early‐onset psychosis

Overall, research suggests that specialist early intervention psychosis services provide better outcomes for young people with psychosis compared with usual mental health service treatment. A systematic review of studies found that specialist early intervention services produced superior outcomes in terms of symptom severity, relapse, remission and recovery, global functioning, involvement in education or employment, and overall quality of life (Correll et al., 2018). What specific factors associated with specialist early intervention are responsible for these superior outcomes requires further research.

### Antipsychotic treatment

Antipsychotic treatment is superior to placebo in early‐onset psychosis (Findling et al., 2008; Haas et al., 2009). A network meta‐analysis found that all antipsychotics that had been tested in placebo‐controlled trials in children and adolescents were effective, with the notable exception of ziprasidone, including aripiprazole, asenapine, lurasidone, olanzapine, quetiapine, and paliperidone (Krause et al., 2018). Meta‐analytic findings show that overall, antipsychotic treatment in adolescents typically leads to moderate effect size improvements in positive symptoms and small effect size improvements in negative symptoms (Ardizzone, Nardecchia, Marconi, Carratelli, & Ferrara, 2010). Olanzapine and risperidone, however, have large effect size benefits over placebo (Correll et al., 2021) and clozapine has large effect size benefits even over olanzapine and risperidone (Krause et al., 2018).

Children and adolescents are more likely to experience side effects, including serious side effects, from antipsychotic treatment compared with adults (Merino et al., 2024). In terms of key adverse effects, most antipsychotics have sedating effects; olanzapine, clozapine, and quetiapine are associated with the greatest weight gain; lurasidone and ziprasidone appear to cause the least weight gain (though, as noted above, ziprasidone is ineffective in adolescent psychosis); and risperidone, haloperidol, paliperidone, and olanzapine are associated with prolactin increase (de Pablo et al., 2024). Whilst olanzapine is a very effective antipsychotic, given its adverse cardiometabolic profile, some guidelines now recommend it be reserved for second‐line use only (Health Service Executive, 2019).

In addition to having a large effect size advantage over other antipsychotics, benefits of clozapine include that it may reduce aggression against both oneself (Desai Boström et al., 2023; Masdrakis & Baldwin, 2023) and others (Kranzler et al., 2005), at least in males. It remains, however, underused as a treatment option in early‐onset psychosis. In the UK, for example, researchers have reported that less than 0.5% of clozapine prescriptions are for patients age <18 (Cirulli, 2005). Research using Danish register data has shown that the time from the first antipsychotic to clozapine use in early‐onset psychosis is in excess of 3 years (Schneider et al., 2015).

Clozapine has important side effects, which must be weighed against its significant benefits. Sedation and hypersalivation are the most common and reported by a large majority of patients with constipation, weight gain, akathisia, and tachycardia also being common (Schneider, Corrigall, Hayes, Kyriakopoulos, & Frangou, 2014). Neutropenia occurs in 6%–15% of adolescents but is usually transient and agranulocytosis is rare (<0.1%) (Schneider et al., 2014). A systematic review did not identify any fatalities associated with clozapine use in early‐onset psychosis (Adnan et al., 2022). When evaluating the tolerability of side effects, it is notable that once commenced, the rate of clozapine discontinuation is low (3%–6%) (Schneider et al., 2014), which is in marked contrast to high rates of discontinuation for most other antipsychotics (Noguera et al., 2013).

### Maintenance antipsychotic treatment

The benefit of maintenance antipsychotic treatment in adolescents with schizophrenia is less clear than for acute treatment. A double‐blind placebo‐controlled maintenance treatment trial of aripiprazole in adolescents with schizophrenia, for example, found that aripiprazole statistically outperformed placebo at 1‐year follow‐up but with a mean difference of just 3–4 points on the total PANSS score (Correll et al., 2017).

A 6‐month (open‐label) risperidone maintenance trial in adolescents with schizophrenia, following on from a 6‐ to 8‐week double‐blind risperidone RCT, found that patients who were maintained on risperidone had a further 11‐point mean total PANSS score reduction at 6‐month follow‐up (Pandina, Kushner, Karcher, & Haas, 2012). Given the maintenance phase did not have a placebo comparison, it is not possible to determine to what extent this improvement can be attributed to risperidone.

A 2‐year open‐label lurasidone maintenance trial in adolescents with schizophrenia, following on from a 6‐week double‐blind lurasidone RCT, found that patients who were maintained on lurasidone had a further 18‐point mean total PANSS score reduction at 2‐year follow‐up (Correll et al., 2022). As above, given the maintenance phase did not include a placebo comparison, it is not possible to determine to what extent this improvement can be attributed to lurasidone.

A 2‐year follow‐up of clozapine treatment in adolescents with schizophrenia found that patients continued to improve after acute treatment, including seeing improvements in 56% of patients who had not had an adequate response at 6 weeks (Adnan et al., 2022; Sporn et al., 2007). This included reduced hospitalisations, with the mean annual hospitalisation rate falling from 0.36 per year to 0.08 per year. For a review of clozapine treatment in early‐onset psychosis, see (Adnan et al., 2022).

There is very limited evidence around the use of long‐acting injectable (LAI) antipsychotics in children and adolescents, mainly comprising case reports and case series. This has included risperidone long‐acting injection, paliperidone palmitate, fluphenazine decanoate, aripiprazole extended‐release injectable, olanzapine extended release, and zuclopenthixol decanoate. A systematic review of the (limited) literature concluded that LAIs may improve clinical outcomes and adherence in early‐onset schizophrenia but also highlighted the substantial methodological limitations of existing studies (Lytle, McVoy, & Sajatovic, 2017). Further well‐conducted trials are needed in this area.

Further research is also needed to determine the optimal duration of antipsychotic treatment in adolescents and to determine who should continue versus discontinue antipsychotic treatment after acute treatment and stabilisation. It is also notable that most clinical trial evidence is based on early‐onset schizophrenia (rather than broader early‐onset psychosis) diagnoses. Further research is necessary to investigate whether the same findings apply to broader psychotic disorders, which are typically not as severe as (narrower) schizophrenia. Regardless, for young people who are prescribed antipsychotics, high‐quality prescribing practices are an important part of both treatment and side effect management. A number of key principles for quality prescribing in early psychosis have been proposed by (Scott et al., 2022), including:

1. Medication choice being informed by adverse effects
2. Close monitoring of metabolic side effects from the outset
3. Regular medication risk–benefit assessment
4. Early consideration of long‐acting injectable (LAI) formulations (though noting, as above, the limited evidence for LAIs specifically in children and adolescents)
5. Assessment of and intervention for co‐occurring mood disorders and
6. Early consideration of clozapine

### Psychosocial interventions

The evidence base on psychosocial interventions for psychosis in children and adolescents is lacking. A recent systematic review of CBT, family intervention, psychoeducation, and cognitive remediation therapy (CRT) in early‐onset psychosis found that none of these interventions significantly improved psychotic symptoms (Anagnostopoulou, Kyriakopoulos, & Alba, 2019). There is evidence that CRT may improve some aspects of cognitive performance (Wykes et al., 2007), but the evidence is of very low certainty [see Daruvala, Kumar, and Datta (2021) and Anagnostopoulou et al. (2019) for reviews].

To date, there have been two studies to specifically investigate CBT in adolescents with psychosis: one in Germany (Müller et al., 2020) and one in the UK (Morrison et al., 2020), as well as one secondary analysis of a CBT trial investigating effectiveness in individuals aged <21 versus 21 years+ (Haddock et al., 2006).

Müller et al. compared treatment as usual (TAU) with CBT plus TAU in adolescents with psychosis. The psychological intervention arm involved individual CBT delivered weekly for 4 weeks and then fortnightly for a further 32 weeks. Treatment was delivered by clinical psychologists with ‘a high level of expertise in the treatment of psychotic disorders’, including specific training in the delivery of CBT for psychosis to adolescents and experience delivering CBT for psychosis in a related research programme. In addition to the one‐to‐one work with adolescents, five sessions were also offered to parents or carers. At the study endpoint, as well as at 24 month follow‐up, the researchers did not find a significant difference between treatment groups in the main study outcome [change in the positive subscale of the Positive and Negative Syndrome Scale (PANSS)] or on overall functioning.

The managing adolescent first‐episode psychosis (MAPS) study (Morrison et al., 2020) compared antipsychotic TAU with psychological intervention either in combination with antipsychotic TAU or psychological intervention alone. The psychological intervention arm involved weekly individual CBT sessions for 6 months delivered by qualified therapists who received weekly supervision and fidelity testing. Regular communication with family members following CBT sessions was built into the protocol, and an additional four booster sessions were provided following the 6‐month treatment. In addition, the psychological treatment arm offered monthly family intervention sessions, based on the behavioural family therapy approach, involving psychoeducation, recovery‐oriented information, problem‐solving, and relapse prevention planning. There was a high rate of adherence to psychological therapy over the course of the trial.

The main outcome, ‘a good response’, was defined as a ≥50% improvement in symptoms on PANSS total score. Despite the intensive programme in the psychological treatment arm, just five of the 16 patients followed up at 6 months (31%) and just three of the 11 patients followed up at 12 months (27%) had achieved a good response. Neither psychological intervention nor combined antipsychotic and psychological treatment outperformed antipsychotic TAU (though noting it was powered only to be a feasibility study, not a superiority trial). Whilst the results of these two trials are disappointing, the high rigour of the studies and intensive expert psychological input is notable. Whilst both trials were considered pilot studies, the poor outcomes for adolescents with psychosis, even with the intensive and highly expert level of psychological input involved in both trials, do not bode well for the effectiveness of CBT in real‐world clinical services for children and adolescents with psychosis. It is also important to consider one additional study, which, in contrast to these two trials, involved an active comparator control.

Haddock et al. carried out secondary analyses of a CBT trial for first‐episode psychosis compared with an active control, supportive psychotherapy (Haddock et al., 2006). Of 309 total participants, 71 were aged <21 years. The researchers found that whilst individuals aged >21 had an overall benefit from CBT, this was not the case for patients aged <21. On multiple measures, the younger cohort had *poorer* outcomes with CBT compared with supportive psychotherapy. With the caveat that these were secondary analyses, these results highlight potentially important developmental differences in treatment efficacy in children and adolescents with psychosis. These findings have received insufficient attention both in clinical guidelines and in the research agenda. This is an important area for future research.

Research on family interventions, one of the pillars of early intervention in psychosis services, is also lacking in early‐onset psychosis. This is, perhaps, surprising given that most children and adolescents with psychosis will live at home with their families and so, the role of the family in managing the illness should be given particularly close inspection. The MAPS study (Morrison et al., 2020), as described above, included the option of family intervention but, because of the design of the study, it was not possible to evaluate its effects separately from CBT.

One other study has looked at a family intervention in adolescents but in individuals at clinical high risk of psychosis, rather than with a psychotic disorder (Miklowitz et al., 2014). Specifically, this study investigated family‐focussed therapy, which was a 6‐month, 18‐session family intervention adapted for individuals at clinical high risk for psychosis, which they compared with an active control (three sessions of psychoeducation). Contrary to their hypotheses (and contrary to the findings for the adults enrolled in the study aged 20–35 years), the researchers found that family intervention was either ineffective (for 12–15‐year olds) or was associated with poorer outcomes (for 16–19‐year olds), compared with the active control.

Taken together, these findings on CBT and family intervention highlight the need to consider possible developmental effects in psychosis treatment, as well as the importance of not making the assumption that psychosocial interventions could not be harmful to adolescents with psychosis. Further research is needed to guide psychosocial treatments for early‐onset psychosis, including research looking beyond CBT. This includes the need for research on interventions to support school/educational needs, which are often severely impacted by psychosis.

Overall, there is a lack of evidence to guide psychosocial interventions in early‐onset psychosis and much of the small body of existing evidence has low or very low reliability. Well‐powered clinical trials of psychosocial interventions, beyond just CBT and family interventions, are sorely needed for early‐onset psychosis.

## Conclusions and future directions

This review has addressed several broad aspects of psychosis in children and adolescents, from psychotic experiences in community and clinical samples to high‐risk clinical groups, to early‐onset psychotic disorders. There have been major advances in our understanding of the prevalence and clinical significance of psychotic experiences/symptoms in community and clinical samples over the past two decades. The question of intervention in this population requires further study, bearing in mind that psychotic experiences are not always associated with psychopathology but that where they are, they tend to be associated with more severe, multimorbid, functionally impairing mental illness.

Further research is needed to capture and refine risk for psychosis in high‐risk groups, as well as further intervention research aimed at reducing this risk. Although around 90% of psychotic disorders have onset after age 18, the recent finding that up to half of psychosis cases diagnosed in the population occurred in individuals who had attended CAP services (age < 18) highlights exciting opportunities to identify risk earlier in life and in far higher numbers than has been possible to date. Research is necessary to identify interventions that might reduce psychosis risk in this population.

Further intervention studies are also needed for children and adolescents diagnosed with psychotic disorders. Most tested antipsychotics have been shown to be effective in acute early‐onset psychosis, although their effect sizes vary, and there are important unanswered questions about maintenance treatment in children and adolescents, which require further research. Evidence from routine, real‐world services shows that overall outcomes from TAU are unacceptably poor.

There is very little evidence to support any specific psychosocial interventions in early‐onset psychosis and, indeed, some limited evidence that there may be important developmental differences between children and adults in terms of what might be helpful and what might be harmful. Further research is needed to assess psychosocial interventions in early‐onset psychosis.

Importantly, these findings illustrate the dangers of simply adopting and applying evidence from adult psychosis studies into child and adolescent settings without appropriate paediatric research and highlight the need for developmentally sensitive healthcare services and healthcare policies for psychosis.

**Key points**

- Psychotic experiences, such as hearing voices or sounds others do not, are common in childhood and are often transient but are associated with an increased risk of the full spectrum of mental health disorders and an increased risk of suicidal behaviour.
- Psychotic disorders are an uncommon outcome for children with psychotic experiences – these experiences are far more frequently associated with common mental disorders, such as depressive, and anxiety disorders, as well as neurodevelopmental disorders.
- There are no indicated treatments for psychotic experiences in and of themselves – if they are a source of distress, the focus for clinicians should typically be on identifying if there are underlying mental health problems or other stressors (which may be contributing to the emergence or persistence of psychotic experiences) and addressing these.
- In early‐onset (age <18) schizophrenia, evidence supports the efficacy of aripiprazole, asenapine, lurasidone, olanzapine, paliperidone, quetiapine, and clozapine during periods of acute psychosis. The evidence is not clear on the optimal duration of subsequent (maintenance) antipsychotic treatment in adolescents.
- There is a lack of evidence to support the effectiveness of psychological treatments in early‐onset schizophrenia, including cognitive behavioural therapy and family therapy, and there has been insufficient research to investigate potential harms associated with psychological interventions.
- Evidence does not support the validity of the clinical high‐risk (CHR) approach in children and adolescents – it is not clear if meeting CHR criteria provides any additional information about psychosis risk in children beyond the risk captured simply by presenting to mental health services in childhood.
- There are currently no evidence‐based interventions to reduce psychosis risk in children and adolescents but this is an area of active research.

## Data Availability

No new data were generated.

## References

[jcpp14088-bib-0001] Addington, J. , & Heinssen, R. (2012). Prediction and prevention of psychosis in youth at clinical high risk. Annual Review of Clinical Psychology, 8, 269–289. 10.1146/annurev-clinpsy-032511-143146 22224837

[jcpp14088-bib-0002] Addington, J. , Piskulic, D. , Liu, L. , Lockwood, J. , Cadenhead, K.S. , Cannon, T.D. , … & Seidman, L.J. (2017). Comorbid diagnoses for youth at clinical high risk of psychosis. Schizophrenia Research, 190, 90–95. 28372906 10.1016/j.schres.2017.03.043 PMC5731830

[jcpp14088-bib-0003] Adnan, M. , Motiwala, F. , Trivedi, C. , Sultana, T. , Mansuri, Z. , & Jain, S. (2022). Clozapine for management of childhood and adolescent‐onset schizophrenia: A systematic review and meta‐analysis. Journal of Child and Adolescent Psychopharmacology, 32, 2–11. 35099269 10.1089/cap.2021.0092

[jcpp14088-bib-0004] Ajnakina, O. , Morgan, C. , Gayer‐Anderson, C. , Oduola, S. , Bourque, F. , Bramley, S. , … & Murray, R.M. (2017). Only a small proportion of patients with first episode psychosis come via prodromal services: A retrospective survey of a large UK mental health programme. BMC Psychiatry, 17, 1–9. 28841826 10.1186/s12888-017-1468-y PMC5574213

[jcpp14088-bib-0005] Anagnostopoulou, N. , Kyriakopoulos, M. , & Alba, A. (2019). Psychological interventions in psychosis in children and adolescents: A systematic review. European Child and Adolescent Psychiatry, 28, 735–746. 29728871 10.1007/s00787-018-1159-3

[jcpp14088-bib-0006] Ardizzone, I. , Nardecchia, F. , Marconi, A. , Carratelli, T.I. , & Ferrara, M. (2010). Antipsychotic medication in adolescents suffering from schizophrenia: A meta‐analysis of randomized controlled trials. Psychopharmacology Bulletin, 43, 45–66. 21052042

[jcpp14088-bib-0007] Baeza, I. , de la Serna, E. , Amoretti, S. , Cuesta, M.J. , Díaz‐Caneja, C.M. , Mezquida, G. , … & Vieta, E. (2021). Premorbid characteristics as predictors of early onset versus adult onset in patients with a first episode of psychosis. The Journal of Clinical Psychiatry, 82, 36672. 10.4088/JCP.21m13907 34529899

[jcpp14088-bib-0008] Bak, M. , Myin‐Germeys, I. , Delespaul, P. , Vollebergh, W. , de Graaf, R. , & van Os, J. (2005). Do different psychotic experiences differentially predict need for care in the general population? Comprehensive Psychiatry, 46, 192–199. 16021589 10.1016/j.comppsych.2004.08.003

[jcpp14088-bib-0009] Bartels‐Velthuis, A.A. , van de Willige, G. , Jenner, J.A. , van Os, J. , & Wiersma, D. (2011). Course of auditory vocal hallucinations in childhood: 5‐year follow‐up study. The British Journal of Psychiatry, 199, 296–302. 21708881 10.1192/bjp.bp.110.086918

[jcpp14088-bib-0010] Bhavsar, V. , Maccabe, J.H. , Hatch, S.L. , Hotopf, M. , Boydell, J. , & McGuire, P. (2017). Subclinical psychotic experiences and subsequent contact with mental health services. BJPsych Open, 3, 64–70. 28357132 10.1192/bjpo.bp.117.004689 PMC5339598

[jcpp14088-bib-0011] Burke, T. , Thompson, A. , Mifsud, N. , Yung, A.R. , Nelson, B. , McGorry, P. , & O'Donoghue, B. (2022). Proportion and characteristics of young people in a first‐episode psychosis clinic who first attended an at‐risk mental state service or other specialist youth mental health service. Schizophrenia Research, 241, 94–101. 35101839 10.1016/j.schres.2021.12.035

[jcpp14088-bib-0012] Catalan, A. , Salazar de Pablo, G. , Vaquerizo Serrano, J. , Mosillo, P. , Baldwin, H. , Fernández‐Rivas, A. , … & Bonoldi, I. (2021). Annual research review: Prevention of psychosis in adolescents–systematic review and meta‐analysis of advances in detection, prognosis and intervention. Journal of Child Psychology and Psychiatry, 62, 657–673. 32924144 10.1111/jcpp.13322

[jcpp14088-bib-0013] Cederlöf, M. , Kuja‐Halkola, R. , Larsson, H. , Sjölander, A. , Östberg, P. , Lundström, S. , … & Lichtenstein, P. (2017). A longitudinal study of adolescent psychotic experiences and later development of substance use disorder and suicidal behavior. Schizophrenia Research, 181, 13–16. 27615409 10.1016/j.schres.2016.08.029

[jcpp14088-bib-0014] Chisholm, K. , Lin, A. , Abu‐Akel, A. , & Wood, S.J. (2015). The association between autism and schizophrenia spectrum disorders: A review of eight alternate models of co‐occurrence. Neuroscience and Biobehavioral Reviews, 55, 173–183. 25956249 10.1016/j.neubiorev.2015.04.012

[jcpp14088-bib-0015] Cirulli, G. (2005). Clozapine prescribing in adolescent psychiatry: Survey of prescribing practice in in‐patient units. Psychiatric Bulletin, 29, 377–380.

[jcpp14088-bib-0016] Clarke, M.C. , Harley, M. , & Cannon, M. (2006). The role of obstetric events in schizophrenia. Schizophrenia Bulletin, 32, 3–8. 16306181 10.1093/schbul/sbj028 PMC2632192

[jcpp14088-bib-0017] Cleverley, K. , Foussias, G. , Ameis, S.H. , Courtney, D.B. , Goldstein, B.I. , Hawke, L.D. , … & Wheeler, A.L. (2023). The Toronto adolescent and youth cohort study: Study design and early data related to psychosis spectrum symptoms, functioning, and suicidality. Biological Psychiatry: Cognitive Neuroscience and Neuroimaging, 9, 253–264. 37979943 10.1016/j.bpsc.2023.10.011

[jcpp14088-bib-0018] Conrad, A.M. , Lewin, T.J. , Sly, K.A. , Schall, U. , Halpin, S.A. , Hunter, M. , & Carr, V.J. (2017). Utility of risk‐status for predicting psychosis and related outcomes: Evaluation of a 10‐year cohort of presenters to a specialised early psychosis community mental health service. Psychiatry Research, 247, 336–344. 27984822 10.1016/j.psychres.2016.12.005

[jcpp14088-bib-0019] Correll, C.U. , Cortese, S. , Croatto, G. , Monaco, F. , Krinitski, D. , Arrondo, G. , … & Estradé, A. (2021). Efficacy and acceptability of pharmacological, psychosocial, and brain stimulation interventions in children and adolescents with mental disorders: An umbrella review. World Psychiatry, 20, 244–275. 34002501 10.1002/wps.20881 PMC8129843

[jcpp14088-bib-0020] Correll, C.U. , Findling, R.L. , Tocco, M. , Pikalov, A. , Deng, L. , & Goldman, R. (2022). Safety and effectiveness of lurasidone in adolescents with schizophrenia: Results of a 2‐year, open‐label extension study‐‐CORRIGENDUM. CNS Spectrums, 27, 129. 33998439 10.1017/S1092852921000511

[jcpp14088-bib-0021] Correll, C.U. , Galling, B. , Pawar, A. , Krivko, A. , Bonetto, C. , Ruggeri, M. , … & Hui, C.L. (2018). Comparison of early intervention services vs treatment as usual for early‐phase psychosis: A systematic review, meta‐analysis, and meta‐regression. JAMA Psychiatry, 75, 555–565. 29800949 10.1001/jamapsychiatry.2018.0623 PMC6137532

[jcpp14088-bib-0022] Correll, C.U. , Kohegyi, E. , Zhao, C. , Baker, R.A. , McQuade, R. , Salzman, P.M. , … & Carson, W. (2017). Oral aripiprazole as maintenance treatment in adolescent schizophrenia: Results from a 52‐week, randomized, placebo‐controlled withdrawal study. Journal of the American Academy of Child & Adolescent Psychiatry, 56, 784–792. 28838583 10.1016/j.jaac.2017.06.013

[jcpp14088-bib-0023] Crush, E. , Arseneault, L. , Moffitt, T.E. , Danese, A. , Caspi, A. , Jaffee, S.R. , … & Fisher, H.L. (2018). Protective factors for psychotic experiences amongst adolescents exposed to multiple forms of victimization. Journal of Psychiatric Research, 104, 32–38. 29929082 10.1016/j.jpsychires.2018.06.011 PMC6109202

[jcpp14088-bib-0024] Dalsgaard, S. , Mortensen, P.B. , Frydenberg, M. , Maibing, C.M. , Nordentoft, M. , & Thomsen, P. (2014). Association between attention‐deficit hyperactivity disorder in childhood and schizophrenia later in adulthood. European Psychiatry, 29, 259–263. 24016863 10.1016/j.eurpsy.2013.06.004

[jcpp14088-bib-0025] Daruvala, R. , Kumar, A. , & Datta, S.S. (2021). Do psychological interventions work for psychosis in adolescents? Schizophrenia Bulletin, 47, 692–694. 32976579 10.1093/schbul/sbaa132 PMC8084436

[jcpp14088-bib-0026] Davies, C. , Cipriani, A. , Ioannidis, J.P. , Radua, J. , Stahl, D. , Provenzani, U. , … & Fusar‐Poli, P. (2018). Lack of evidence to favor specific preventive interventions in psychosis: A network meta‐analysis. World Psychiatry, 17, 196–209. 29856551 10.1002/wps.20526 PMC5980552

[jcpp14088-bib-0027] Davies, S.E. , Neufeld, S.A. , van Sprang, E. , Schweren, L. , Keivit, R. , Fonagy, P. , … & Reynolds, S. (2020). Trajectories of depression symptom change during and following treatment in adolescents with unipolar major depression. Journal of Child Psychology and Psychiatry, 61, 565–574. 31647124 10.1111/jcpp.13145 PMC7216986

[jcpp14088-bib-0028] de Jong, Y. , Mulder, C.L. , Boon, A.E. , Deen, M. , van't Hof, M. , & van der Gaag, M. (2018). Screening for psychosis risk among adolescents in child and adolescent mental health services: A description of the first step with the 16‐item version of the prodromal questionnaire (PQ‐16). Early Intervention in Psychiatry, 12, 669–676. 27860294 10.1111/eip.12362

[jcpp14088-bib-0029] De Pablo, G.S. , Radua, J. , Pereira, J. , Bonoldi, I. , Arienti, V. , Besana, F. , … & Catalan, A. (2021). Probability of transition to psychosis in individuals at clinical high risk: An updated meta‐analysis. JAMA Psychiatry, 78, 970–978. 34259821 10.1001/jamapsychiatry.2021.0830 PMC8281006

[jcpp14088-bib-0030] de Pablo, G.S. , Rodriguez, V. , Besana, F. , Civardi, S.C. , Arienti, V. , Garceo, L.M. , … & Abbott, C. (2024). Umbrella review: Early‐onset psychosis: Atlas of the meta‐analytical evidence. Journal of the American Academy of Child & Adolescent Psychiatry. 10.1016/j.jaac.2023.10.016 38280414

[jcpp14088-bib-0031] De Pablo, G.S. , Soardo, L. , Cabras, A. , Pereira, J. , Kaur, S. , Besana, F. , … & Solmi, M. (2022). Clinical outcomes in individuals at clinical high risk of psychosis who do not transition to psychosis: A meta‐analysis. Epidemiology and Psychiatric Sciences, 31, e9. 35042573 10.1017/S2045796021000639 PMC8786617

[jcpp14088-bib-0032] Desai Boström, A.E. , Andersson, P. , Rask‐Andersen, M. , Jarbin, H. , Lundberg, J. , & Jokinen, J. (2023). Regional clozapine, ECT and lithium usage inversely associated with excess suicide rates in male adolescents. Nature Communications, 14, 1281. 10.1038/s41467-023-36973-4 PMC10015020 36918566

[jcpp14088-bib-0033] DeVylder, J. , & Kelleher, I. (2016). Clinical significance of psychotic experiences in the context of sleep disturbance or substance use. Psychological Medicine, 46, 1761–1767. 26951467 10.1017/S0033291716000271

[jcpp14088-bib-0034] DeVylder, J.E. , Lukens, E.P. , Link, B.G. , & Lieberman, J.A. (2015). Suicidal ideation and suicide attempts among adults with psychotic experiences: Data from the collaborative psychiatric epidemiology surveys. JAMA Psychiatry, 72, 219–225. 25715312 10.1001/jamapsychiatry.2014.2663

[jcpp14088-bib-0035] Dhossche, D. , Ferdinand, R. , van der Ende, J. , Hofstra, M. , & Verhulst, F. (2002). Diagnostic outcome of self‐reported hallucinations in a community sample of adolescents. Psychological Medicine, 32, 619–627. 12102376 10.1017/s003329170200555x

[jcpp14088-bib-0036] Dominguez, M. , Wichers, M. , Lieb, R. , Wittchen, H.‐U. , & van Os, J. (2011). Evidence that onset of clinical psychosis is an outcome of progressively more persistent subclinical psychotic experiences: An 8‐year cohort study. Schizophrenia Bulletin, 37, 84–93. 19460881 10.1093/schbul/sbp022 PMC3004179

[jcpp14088-bib-0037] Ellersgaard, D. , Gregersen, M. , Spang, K.S. , Christiani, C. , Burton, B.K. , Hemager, N. , … & Jepsen, J.R.M. (2021). Psychotic experiences in seven‐year‐old children with familial high risk of schizophrenia or bipolar disorder in: The Danish high risk and resilience study–VIA 7; A population‐based cohort study. Schizophrenia Research, 228, 510–518. 33308959 10.1016/j.schres.2020.11.045

[jcpp14088-bib-0038] Findling, R.L. , Robb, A. , Nyilas, M. , Forbes, R.A. , Jin, N. , Ivanova, S. , … & Carson, W.H. (2008). A multiple‐center, randomized, double‐blind, placebo‐controlled study of oral aripiprazole for treatment of adolescents with schizophrenia. American Journal of Psychiatry, 165, 1432–1441. 18765484 10.1176/appi.ajp.2008.07061035

[jcpp14088-bib-0039] Fisher, H. , Caspi, A. , Poulton, R. , Meier, M. , Houts, R. , Harrington, H. , … & Moffitt, T. (2013). Specificity of childhood psychotic symptoms for predicting schizophrenia by 38 years of age: A birth cohort study. Psychological Medicine, 43, 2077–2086. 23302254 10.1017/S0033291712003091 PMC3758773

[jcpp14088-bib-0040] Freeman, D. , Sheaves, B. , Goodwin, G.M. , Yu, L.‐M. , Nickless, A. , Harrison, P.J. , … & Wadekar, V. (2017). The effects of improving sleep on mental health (OASIS): A randomised controlled trial with mediation analysis. The Lancet Psychiatry, 4, 749–758. 28888927 10.1016/S2215-0366(17)30328-0 PMC5614772

[jcpp14088-bib-0041] Ganesh, S. , & D'Souza, D.C. (2022). Cannabis and psychosis: Recent epidemiological findings continuing the “causality debate”. American Psychiatric Association, 179, 8–10. 10.1176/appi.ajp.2021.21111126 34974754

[jcpp14088-bib-0042] Goldstein, J.M. , Buka, S.L. , Seidman, L.J. , & Tsuang, M.T. (2010). Specificity of familial transmission of schizophrenia psychosis spectrum and affective psychoses in the New England family study's high‐risk design. Archives of General Psychiatry, 67, 458–467. 20439827 10.1001/archgenpsychiatry.2010.38 PMC3049996

[jcpp14088-bib-0043] Gomes, F.V. , Rincón‐Cortés, M. , & Grace, A.A. (2016). Adolescence as a period of vulnerability and intervention in schizophrenia: Insights from the MAM model. Neuroscience and Biobehavioral Reviews, 70, 260–270. 27235082 10.1016/j.neubiorev.2016.05.030 PMC5074867

[jcpp14088-bib-0044] Gregersen, M. , Møllegaard Jepsen, J.R. , Rohd, S.B. , Søndergaard, A. , Brandt, J.M. , Ellersgaard, D. , … & Wilms, M. (2022). Developmental pathways and clinical outcomes of early childhood psychotic experiences in preadolescent children at familial high risk of schizophrenia or bipolar disorder: A prospective, longitudinal cohort study‐the Danish high risk and resilience study, VIA 11. American Journal of Psychiatry, 179, 628–639. 36048497 10.1176/appi.ajp.21101076

[jcpp14088-bib-0045] Haas, M. , Unis, A.S. , Armenteros, J. , Copenhaver, M.D. , Quiroz, J.A. , & Kushner, S.F. (2009). A 6‐week, randomized, double‐blind, placebo‐controlled study of the efficacy and safety of risperidone in adolescents with schizophrenia. Journal of Child and Adolescent Psychopharmacology, 19, 611–621. 20035579 10.1089/cap.2008.0144

[jcpp14088-bib-0046] Haddock, G. , Lewis, S. , Bentall, R. , Dunn, G. , Drake, R. , & Tarrier, N. (2006). Influence of age on outcome of psychological treatments in first‐episode psychosis. The British Journal of Psychiatry, 188, 250–254. 16507967 10.1192/bjp.188.3.250

[jcpp14088-bib-0047] Health Service Executive . (2019). National clinical programme for early intervention in psychosis . https://www.hse.ie/eng/about/who/cspd/ncps/mental‐health/psychosis/resources/hse‐early‐intervention‐in‐psychosis‐model‐of‐care‐june‐20191.pdf

[jcpp14088-bib-0048] Healy, C. , Brannigan, R. , Dooley, N. , Coughlan, H. , Clarke, M. , Kelleher, I. , & Cannon, M. (2019). Childhood and adolescent psychotic experiences and risk of mental disorder: A systematic review and meta‐analysis. Psychological Medicine, 49, 1589–1599. 31088578 10.1017/S0033291719000485

[jcpp14088-bib-0049] Healy, C. , Lång, U. , O'Hare, K. , Veijola, J. , O'Connor, K. , Lahti‐Pulkkinen, M. , … & Kelleher, I. (2024). Sensitivity of the familial high‐risk approach for the prediction of future psychosis: A total population study. World Psychiatry, 23, 432–437. 39279372 10.1002/wps.21243 PMC11403180

[jcpp14088-bib-0050] Hielscher, E. , DeVylder, J. , Connell, M. , Hasking, P. , Martin, G. , & Scott, J.G. (2020). Investigating the role of hallucinatory experiences in the transition from suicidal thoughts to attempts. Acta Psychiatrica Scandinavica, 141, 241–253. 31721142 10.1111/acps.13128

[jcpp14088-bib-0051] Hielscher, E. , DeVylder, J. , Hasking, P. , Connell, M. , Martin, G. , & Scott, J.G. (2021). Can't get you out of my head: Persistence and remission of psychotic experiences in adolescents and its association with self‐injury and suicide attempts. Schizophrenia Research, 229, 63–72. 33248885 10.1016/j.schres.2020.11.019

[jcpp14088-bib-0052] Hjorthøj, C. , Posselt, C.M. , & Nordentoft, M. (2021). Development over time of the population‐attributable risk fraction for cannabis use disorder in schizophrenia in Denmark. JAMA Psychiatry, 78, 1013–1019. 34287621 10.1001/jamapsychiatry.2021.1471 PMC8295899

[jcpp14088-bib-0053] Howes, O. , & McCutcheon, R. (2017). Inflammation and the neural diathesis‐stress hypothesis of schizophrenia: A reconceptualization. Translational Psychiatry, 7, e1024. 28170004 10.1038/tp.2016.278 PMC5438023

[jcpp14088-bib-0054] Hui, C. , Morcillo, C. , Russo, D.A. , Stochl, J. , Shelley, G.F. , Painter, M. , … & Perez, J. (2013). Psychiatric morbidity, functioning and quality of life in young people at clinical high risk for psychosis. Schizophrenia Research, 148, 175–180. 23773297 10.1016/j.schres.2013.05.026 PMC3744805

[jcpp14088-bib-0055] Insel, T.R. (2010). Rethinking schizophrenia. Nature, 468, 187–193. 21068826 10.1038/nature09552

[jcpp14088-bib-0056] Ioakeimidis, V. , Haenschel, C. , Yarrow, K. , Kyriakopoulos, M. , & Dima, D. (2020). A meta‐analysis of structural and functional brain abnormalities in early‐onset schizophrenia. Schizophrenia Bulletin Open, 1, sgaa016.

[jcpp14088-bib-0057] Johns, L.C. , & van Os, J. (2001). The continuity of psychotic experiences in the general population. Clinical Psychology Review, 21, 1125–1141. 11702510 10.1016/s0272-7358(01)00103-9

[jcpp14088-bib-0058] Johnstone, E.C. , Lawrie, S.M. , & Cosway, R. (2002). What does the Edinburgh high‐risk study tell us about schizophrenia? American Journal of Medical Genetics, 114, 906–912. 12457384 10.1002/ajmg.b.10304

[jcpp14088-bib-0059] Kääriälä, A. , Gyllenberg, D. , Sund, R. , Pekkarinen, E. , Keski‐Säntti, M. , Ristikari, T. , … & Sourander, A. (2022). The association between treated psychiatric and neurodevelopmental disorders and out‐of‐home care among Finnish children born in 1997. European Child and Adolescent Psychiatry, 31, 1789–1798. 34101021 10.1007/s00787-021-01819-1 PMC9666323

[jcpp14088-bib-0060] Karlsgodt, K.H. (2023). Early risk factors in early‐onset psychosis. In Adolescent psychosis (pp. 81–105). Cambridge, Massachussetts: Elsevier.

[jcpp14088-bib-0061] Kelleher, I. (2023). Psychosis prediction 2.0: Why child and adolescent mental health services should be a key focus for schizophrenia and bipolar disorder prevention research. The British Journal of Psychiatry, 222, 185–187. 36632815 10.1192/bjp.2022.193

[jcpp14088-bib-0062] Kelleher, I. , Connor, D. , Clarke, M.C. , Devlin, N. , Harley, M. , & Cannon, M. (2012). Prevalence of psychotic symptoms in childhood and adolescence: A systematic review and meta‐analysis of population‐based studies. Psychological Medicine, 42, 1857–1863. 22225730 10.1017/S0033291711002960

[jcpp14088-bib-0063] Kelleher, I. , Corcoran, P. , Keeley, H. , Wigman, J.T. , Devlin, N. , Ramsay, H. , … & Hoven, C. (2013). Psychotic symptoms and population risk for suicide attempt: A prospective cohort study. JAMA Psychiatry, 70, 940–948. 23863946 10.1001/jamapsychiatry.2013.140

[jcpp14088-bib-0064] Kelleher, I. , Devlin, N. , Wigman, J.T. , Kehoe, A. , Murtagh, A. , Fitzpatrick, C. , & Cannon, M. (2014). Psychotic experiences in a mental health clinic sample: Implications for suicidality, multimorbidity and functioning. Psychological Medicine, 44, 1615–1624. 24025687 10.1017/S0033291713002122

[jcpp14088-bib-0065] Kelleher, I. , Keeley, H. , Corcoran, P. , Lynch, F. , Fitzpatrick, C. , Devlin, N. , … & Harley, M. (2012). Clinicopathological significance of psychotic experiences in non‐psychotic young people: Evidence from four population‐based studies. The British Journal of Psychiatry, 201, 26–32. 22500011 10.1192/bjp.bp.111.101543

[jcpp14088-bib-0066] Kincaid, D.L. , Doris, M. , Shannon, C. , & Mulholland, C. (2017). What is the prevalence of autism spectrum disorder and ASD traits in psychosis? A systematic review. Psychiatry Research, 250, 99–105. 28152400 10.1016/j.psychres.2017.01.017

[jcpp14088-bib-0067] Knight, C. , Russo, D. , Stochl, J. , Croudace, T. , Fowler, D. , Grey, N. , … & Perez, J. (2020). Prevalence of and recovery from common mental disorder including psychotic experiences in the UK primary care improving access to psychological therapies (IAPT) programme. Journal of Affective Disorders, 272, 84–90. 32379625 10.1016/j.jad.2020.04.015

[jcpp14088-bib-0068] Kranzler, H. , Roofeh, D. , Gerbino‐Rosen, G. , Dombrowski, C. , McMeniman, M. , DeThomas, C. , … & Fisch, G.S. (2005). Clozapine: Its impact on aggressive behavior among children and adolescents with schizophrenia. Journal of the American Academy of Child & Adolescent Psychiatry, 44, 55–63. 15608544 10.1097/01.chi.0000145371.23122.5a

[jcpp14088-bib-0069] Krause, M. , Zhu, Y. , Huhn, M. , Schneider‐Thoma, J. , Bighelli, I. , Chaimani, A. , & Leucht, S. (2018). Efficacy, acceptability, and tolerability of antipsychotics in children and adolescents with schizophrenia: A network meta‐analysis. European Neuropsychopharmacology, 28, 659–674. 29802039 10.1016/j.euroneuro.2018.03.008

[jcpp14088-bib-0070] Lång, U. , Ramsay, H. , Yates, K. , Veijola, J. , Gyllenberg, D. , Clarke, M.C. , … & Kelleher, I. (2022). Potential for prediction of psychosis and bipolar disorder in child and adolescent mental health services: A longitudinal register study of all people born in Finland in 1987. World Psychiatry, 21(3), 436–443. 36073707 10.1002/wps.21009 PMC9453911

[jcpp14088-bib-0071] Lång, U. , Yates, K. , Leacy, F.P. , Clarke, M.C. , McNicholas, F. , Cannon, M. , & Kelleher, I. (2022). Systematic review and meta‐analysis: Psychosis risk in children and adolescents with an at‐risk mental state. Journal of the American Academy of Child & Adolescent Psychiatry, 61, 615–625. 34363965 10.1016/j.jaac.2021.07.593

[jcpp14088-bib-0072] Lee, J. , Rekhi, G. , Mitter, N. , Bong, Y.L. , Kraus, M.S. , Lam, M. , … & Chong, S.A. (2013). The longitudinal youth at risk study (LYRIKS) – An Asian UHR perspective. Schizophrenia Research, 151, 279–283. 24139196 10.1016/j.schres.2013.09.025

[jcpp14088-bib-0073] Lemmers‐Jansen, I. , Krabbendam, L. , & van der Ven, E. (2023). The epidemiology of early‐onset psychosis. In Adolescent psychosis (pp. 31–50). Cambridge, Massachussetts: Elsevier.

[jcpp14088-bib-0074] Lindgren, M. , Kuvaja, H. , Jokela, M. , & Therman, S. (2023). Predictive validity of psychosis risk models when applied to adolescent psychiatric patients. Psychological Medicine, 53, 547–558. 34024309 10.1017/S0033291721001938

[jcpp14088-bib-0075] Lindgren, M. , Manninen, M. , Kalska, H. , Mustonen, U. , Laajasalo, T. , Moilanen, K. , … & Therman, S. (2014). Predicting psychosis in a general adolescent psychiatric sample. Schizophrenia Research, 158, 1–6. 25015028 10.1016/j.schres.2014.06.028

[jcpp14088-bib-0076] Lytle, S. , McVoy, M. , & Sajatovic, M. (2017). Long‐acting injectable antipsychotics in children and adolescents. Journal of Child and Adolescent Psychopharmacology, 27, 2–9. 28112539 10.1089/cap.2016.0055

[jcpp14088-bib-0077] Maibing, C.F. , Pedersen, C.B. , Benros, M.E. , Mortensen, P.B. , Dalsgaard, S. , & Nordentoft, M. (2015). Risk of schizophrenia increases after all child and adolescent psychiatric disorders: A nationwide study. Schizophrenia Bulletin, 41, 963–970. 25193974 10.1093/schbul/sbu119 PMC4466169

[jcpp14088-bib-0078] Maijer, K. , Hayward, M. , Fernyhough, C. , Calkins, M.E. , Debbané, M. , Jardri, R. , … & Scott, J.G. (2019). Hallucinations in children and adolescents: An updated review and practical recommendations for clinicians. Schizophrenia Bulletin, 45(Supplement_1), S5–S23. 30715540 10.1093/schbul/sby119 PMC6357982

[jcpp14088-bib-0079] Manninen, M. , Lindgren, M. , Therman, S. , Huttunen, M. , Ebeling, H. , Moilanen, I. , & Suvisaari, J. (2014). Clinical high‐risk state does not predict later psychosis in a delinquent adolescent population. Early Intervention in Psychiatry, 8, 87–90. 23575313 10.1111/eip.12045

[jcpp14088-bib-0080] Masdrakis, V.G. , & Baldwin, D.S. (2023). Prevention of suicide by clozapine in mental disorders: Systematic review. European Neuropsychopharmacology, 69, 4–23. 36640481 10.1016/j.euroneuro.2022.12.011

[jcpp14088-bib-0081] McCarthy‐Jones, S. , Krueger, J. , Larøi, F. , Broome, M. , & Fernyhough, C. (2013). Stop, look, listen: The need for philosophical phenomenological perspectives on auditory verbal hallucinations. Frontiers in Human Neuroscience, 7, 127. 23576974 10.3389/fnhum.2013.00127 PMC3620561

[jcpp14088-bib-0082] McCutcheon, R.A. , Marques, T.R. , & Howes, O.D. (2020). Schizophrenia – An overview. JAMA Psychiatry, 77, 201–210. 31664453 10.1001/jamapsychiatry.2019.3360

[jcpp14088-bib-0083] McGlashan, T.H. , Zipursky, R.B. , Perkins, D. , Addington, J. , Miller, T. , Woods, S.W. , … & Epstein, I. (2006). Randomized, double‐blind trial of olanzapine versus placebo in patients prodromally symptomatic for psychosis. American Journal of Psychiatry, 163, 790–799. 16648318 10.1176/ajp.2006.163.5.790

[jcpp14088-bib-0084] McGorry, P.D. , Nelson, B. , Markulev, C. , Yuen, H.P. , Schäfer, M.R. , Mossaheb, N. , … & Berger, G.E. (2017). Effect of ω‐3 polyunsaturated fatty acids in young people at ultrahigh risk for psychotic disorders: The NEURAPRO randomized clinical trial. JAMA Psychiatry, 74, 19–27. 27893018 10.1001/jamapsychiatry.2016.2902

[jcpp14088-bib-0085] McGrath, J.J. , Saha, S. , Al‐Hamzawi, A. , Alonso, J. , Bromet, E.J. , Bruffaerts, R. , … & Fayyad, J. (2015). Psychotic experiences in the general population: A cross‐national analysis based on 31 261 respondents from 18 countries. JAMA Psychiatry, 72, 697–705. 26018466 10.1001/jamapsychiatry.2015.0575 PMC5120396

[jcpp14088-bib-0086] Merino, D. , Gérard, A.O. , Destere, A. , Saidessalam, H. , Askenazy, F. , Montastruc, F. , … & Thümmler, S. (2024). Cardiac and metabolic safety profile of antipsychotics in youths: A WHO safety database analysis. Psychiatry Research, 334, 115786. 38387164 10.1016/j.psychres.2024.115786

[jcpp14088-bib-0087] Michel, C. , Ruhrmann, S. , Schimmelmann, B.G. , Klosterkötter, J. , & Schultze‐Lutter, F. (2018). Course of clinical high‐risk states for psychosis beyond conversion. European Archives of Psychiatry and Clinical Neuroscience, 268, 39–48. 28054132 10.1007/s00406-016-0764-8

[jcpp14088-bib-0088] Miklowitz, D.J. , O'Brien, M.P. , Schlosser, D.A. , Addington, J. , Candan, K.A. , Marshall, C. , … & De Silva, S.D. (2014). Family‐focused treatment for adolescents and young adults at high risk for psychosis: Results of a randomized trial. Journal of the American Academy of Child & Adolescent Psychiatry, 53, 848–858. 25062592 10.1016/j.jaac.2014.04.020 PMC4112074

[jcpp14088-bib-0089] Mongan, D. , Ramesar, M. , Föcking, M. , Cannon, M. , & Cotter, D. (2020). Role of inflammation in the pathogenesis of schizophrenia: A review of the evidence, proposed mechanisms and implications for treatment. Early Intervention in Psychiatry, 14, 385–397. 31368253 10.1111/eip.12859

[jcpp14088-bib-0090] Moran, L.V. , Ongur, D. , Hsu, J. , Castro, V.M. , Perlis, R.H. , & Schneeweiss, S. (2019). Psychosis with methylphenidate or amphetamine in patients with ADHD. New England Journal of Medicine, 380, 1128–1138. 30893533 10.1056/NEJMoa1813751 PMC6543546

[jcpp14088-bib-0091] Morgan, C. , & Fisher, H. (2007). Environment and schizophrenia: Environmental factors in schizophrenia: Childhood trauma – A critical review. Schizophrenia Bulletin, 33, 3–10. 17105965 10.1093/schbul/sbl053 PMC2632300

[jcpp14088-bib-0092] Morrison, A.P. , French, P. , Stewart, S.L. , Birchwood, M. , Fowler, D. , Gumley, A.I. , … & Murray, G.K. (2012). Early detection and intervention evaluation for people at risk of psychosis: Multisite randomised controlled trial. BMJ, 344, e2233. 22491790 10.1136/bmj.e2233 PMC3320714

[jcpp14088-bib-0093] Morrison, A.P. , Pyle, M. , Maughan, D. , Johns, L. , Freeman, D. , Broome, M.R. , … & MacLennan, G. (2020). Antipsychotic medication versus psychological intervention versus a combination of both in adolescents with first‐episode psychosis (MAPS): A multicentre, three‐arm, randomised controlled pilot and feasibility study. The Lancet Psychiatry, 7, 788–800. 32649925 10.1016/S2215-0366(20)30248-0 PMC7606914

[jcpp14088-bib-0094] Mortensen, P.B. , Pedersen, C.B. , Westergaard, T. , Wohlfahrt, J. , Ewald, H. , Mors, O. , … & Melbye, M. (1999). Effects of family history and place and season of birth on the risk of schizophrenia. New England Journal of Medicine, 340, 603–608. 10029644 10.1056/NEJM199902253400803

[jcpp14088-bib-0095] Mortensen, P.B. , Pedersen, M.G. , & Pedersen, C. (2010). Psychiatric family history and schizophrenia risk in Denmark: Which mental disorders are relevant? Psychological Medicine, 40, 201–210. 19607751 10.1017/S0033291709990419

[jcpp14088-bib-0096] Müller, A.D. , Gjøde, I.C.T. , Thams, N. , Ingversen, S. , Moszkowicz, M. , Jepsen, J.R.M. , … & Nordentoft, M. (2024). Family‐based preventive intervention for children of parents with severe mental illness: A randomized clinical trial. JCPP Advances, e12216. 39411478 10.1002/jcv2.12216 PMC11472814

[jcpp14088-bib-0097] Müller, H. , Kommescher, M. , Güttgemanns, J. , Wessels, H. , Walger, P. , Lehmkuhl, G. , … & Bechdolf, A. (2020). Cognitive behavioral therapy in adolescents with early‐onset psychosis: A randomized controlled pilot study. European Child and Adolescent Psychiatry, 29, 1011–1022. 31599351 10.1007/s00787-019-01415-4

[jcpp14088-bib-0098] Murphy, J. , McBride, O. , Fried, E. , & Shevlin, M. (2018). Distress, impairment and the extended psychosis phenotype: A network analysis of psychotic experiences in an US general population sample. Schizophrenia Bulletin, 44, 768–777. 29036519 10.1093/schbul/sbx134 PMC6007708

[jcpp14088-bib-0099] Narita, Z. , Wilcox, H.C. , & DeVylder, J. (2020). Psychotic experiences and suicidal outcomes in a general population sample. Schizophrenia Research, 215, 223–228. 31668492 10.1016/j.schres.2019.10.024

[jcpp14088-bib-0100] Nevriana, A. , Kosidou, K. , Hope, H. , Wicks, S. , Dalman, C. , Pierce, M. , & Abel, K.M. (2024). Parental mental illness and the likelihood of child out‐of‐home care: A cohort study. Pediatrics, 153, e2023061531. 38312009 10.1542/peds.2023-061531

[jcpp14088-bib-0101] Nishida, A. , Tanii, H. , Nishimura, Y. , Kajiki, N. , Inoue, K. , Okada, M. , … & Okazaki, Y. (2008). Associations between psychotic‐like experiences and mental health status and other psychopathologies among Japanese early teens. Schizophrenia Research, 99, 125–133. 18248792 10.1016/j.schres.2007.11.038

[jcpp14088-bib-0102] Noguera, A. , Ballesta, P. , Baeza, I. , Arango, C. , de la Serna, E. , González‐Pinto, A. , … & Otero, S. (2013). Twenty‐four months of antipsychotic treatment in children and adolescents with first psychotic episode: Discontinuation and tolerability. Journal of Clinical Psychopharmacology, 33, 463–471. 23771198 10.1097/JCP.0b013e3182962480

[jcpp14088-bib-0103] Oh, H. , Karcher, N.R. , Soffer‐Dudek, N. , Koyanagi, A. , Besecker, M. , & DeVylder, J.E. (2024). Distress related to psychotic experiences: Enhancing the world health organization composite international diagnostic interview psychosis screen. International Journal of Methods in Psychiatric Research, 33, e1977. 10.1002/mpr.1977 PMC10804262 37194720

[jcpp14088-bib-0104] O'Hare, K. , Fadiloglu, K. , Lång, U. , Healy, C. , Cannon, M. , DeVylder, J. , & Kelleher, I. (2024). Psychotic experiences and risk of suicidal thoughts and behaviors: A systematic review and meta‐analysis of longitudinal population studies. Schizophrenia Bulletin, sbae197. 39550208 10.1093/schbul/sbae197 PMC12809804

[jcpp14088-bib-0105] O'Hare, K.J. , Poulton, R. , & Linscott, R.J. (2021). Psychotic experiences and schizotypy in early adolescence predict subsequent suicidal ideation trajectories and suicide attempt outcomes from age 18 to 38 years. Schizophrenia Bulletin, 47, 456–464. 33085764 10.1093/schbul/sbaa151 PMC7965071

[jcpp14088-bib-0106] Pandina, G. , Kushner, S. , Karcher, K. , & Haas, M. (2012). An open‐label, multicenter evaluation of the long‐term safety and efficacy of risperidone in adolescents with schizophrenia. Child and Adolescent Psychiatry and Mental Health, 6, 1–15. 22676070 10.1186/1753-2000-6-23 PMC3489792

[jcpp14088-bib-0107] Paolicelli, R.C. , Bolasco, G. , Pagani, F. , Maggi, L. , Scianni, M. , Panzanelli, P. , … & Dumas, L. (2011). Synaptic pruning by microglia is necessary for normal brain development. Science, 333, 1456–1458. 21778362 10.1126/science.1202529

[jcpp14088-bib-0108] Pardo, M. , Matalí, J.L. , Sivoli, J. , Regina, V.‐B. , Butjosa, A. , Dolz, M. , … & Baños, I. (2021). Early onset psychosis and cannabis use: Prevalence, clinical presentation and influence of daily use. Asian Journal of Psychiatry, 62, 102714. 34090251 10.1016/j.ajp.2021.102714

[jcpp14088-bib-0109] Patterson, B. , Boyle, M.H. , Kivlenieks, M. , & Van Ameringen, M. (2016). The use of waitlists as control conditions in anxiety disorders research. Journal of Psychiatric Research, 83, 112–120. 27585425 10.1016/j.jpsychires.2016.08.015

[jcpp14088-bib-0110] Paus, T. , Keshavan, M. , & Giedd, J.N. (2008). Why do many psychiatric disorders emerge during adolescence? Nature Reviews Neuroscience, 9, 947–957. 19002191 10.1038/nrn2513 PMC2762785

[jcpp14088-bib-0111] Pelizza, L. , Azzali, S. , Garlassi, S. , Paterlini, F. , Scazza, I. , Chiri, L.R. , … & Raballo, A. (2018). Adolescents at ultra‐high risk of psychosis in Italian neuropsychiatry services: Prevalence, psychopathology and transition rate. European Child and Adolescent Psychiatry, 27, 725–737. 29058115 10.1007/s00787-017-1070-3

[jcpp14088-bib-0112] Perlis, R.H. , Uher, R. , Ostacher, M. , Goldberg, J.F. , Trivedi, M.H. , Rush, A.J. , & Fava, M. (2011). Association between bipolar spectrum features and treatment outcomes in outpatients with major depressive disorder. Archives of General Psychiatry, 68, 351–360. 21135313 10.1001/archgenpsychiatry.2010.179 PMC3794668

[jcpp14088-bib-0113] Poulton, R. , Caspi, A. , Moffitt, T.E. , Cannon, M. , Murray, R. , & Harrington, H. (2000). Children's self‐reported psychotic symptoms and adult schizophreniform disorder: A 15‐year longitudinal study. Archives of General Psychiatry, 57, 1053–1058. 11074871 10.1001/archpsyc.57.11.1053

[jcpp14088-bib-0114] Qurashi, I. , Chaudhry, I.B. , Khoso, A.B. , Omair Husain, M. , Hafeez, D. , Kiran, T. , … & Husain, N. (2024). A randomised double‐blind placebo‐controlled trial of minocycline and/or omega‐3 fatty acids added to treatment as usual for at risk mental states: The NAYAB study. Brain, Behavior, and Immunity, 115, 609–616. 37924960 10.1016/j.bbi.2023.10.025

[jcpp14088-bib-0115] Rimvall, M.K. , van Os, J. , Verhulst, F. , Wolf, R.T. , Larsen, J.T. , Clemmensen, L. , … & Jeppesen, P. (2020). Mental health service use and psychopharmacological treatment following psychotic experiences in preadolescence. American Journal of Psychiatry, 177, 318–326. 32098486 10.1176/appi.ajp.2019.19070724

[jcpp14088-bib-0116] Rimvall, M.K. , Vassard, D. , Christensen, R. , Nielsen, S.M. , Pagsberg, A.K. , Correll, C.U. , & Jeppesen, P. (2024). Do psychotic experiences act as effect modifiers in youths with common mental health problems allocated to transdiagnostic cognitive behavioural therapy versus management as usual? Secondary analyses of the mind‐my‐mind randomized trial. Early Intervention in Psychiatry, 18, 26–33. 37078563 10.1111/eip.13423

[jcpp14088-bib-0117] Roest, A.M. , de Vries, Y.A. , Wienen, A.W. , & de Jonge, P. (2023). Editorial perspective: Are treatments for childhood mental disorders helpful in the long run? An overview of systematic reviews. Journal of Child Psychology and Psychiatry, 64, 464–469. 36038140 10.1111/jcpp.13677 PMC10086958

[jcpp14088-bib-0118] Rosso, I.M. , Cannon, T.D. , Huttunen, T. , Huttunen, M.O. , Lönnqvist, J. , & Gasperoni, T.L. (2000). Obstetric risk factors for early‐onset schizophrenia in a Finnish birth cohort. American Journal of Psychiatry, 157, 801–807. 10784475 10.1176/appi.ajp.157.5.801

[jcpp14088-bib-0119] Rubino, T. , Prini, P. , Piscitelli, F. , Zamberletti, E. , Trusel, M. , Melis, M. , … & Di Marzo, V. (2015). Adolescent exposure to THC in female rats disrupts developmental changes in the prefrontal cortex. Neurobiology of Disease, 73, 60–69. 25281318 10.1016/j.nbd.2014.09.015

[jcpp14088-bib-0120] Saha, S. , Scott, J.G. , Johnston, A.K. , Slade, T.N. , Varghese, D. , Carter, G.L. , & McGrath, J.J. (2011). The association between delusional‐like experiences and suicidal thoughts and behaviour. Schizophrenia Research, 132, 197–202. 21813264 10.1016/j.schres.2011.07.012

[jcpp14088-bib-0121] Sakai, J. (2020). How synaptic pruning shapes neural wiring during development and, possibly, in disease. Proceedings of the National Academy of Sciences, 117, 16096–16099. 10.1073/pnas.2010281117 PMC7368197 32581125

[jcpp14088-bib-0122] Salazar de Pablo, G. , Guinart, D. , Cornblatt, B.A. , Auther, A.M. , Jiménez‐Fernández, S. , Walitza, S. , … & Correll, C.U. (2020). DSM‐5 attenuated psychosis syndrome in adolescents hospitalized with non‐psychotic psychiatric disorders. Frontiers in Psychiatry, 11, 568982. 33192693 10.3389/fpsyt.2020.568982 PMC7609900

[jcpp14088-bib-0123] Schmidt, S.J. , Schultze‐Lutter, F. , Schimmelmann, B.G. , Maric, N.P. , Salokangas, R.K.R. , Riecher‐Rössler, A. , … & Ruhrmann, S. (2015). EPA guidance on the early intervention in clinical high risk states of psychoses. European Psychiatry, 30, 388–404. 25749390 10.1016/j.eurpsy.2015.01.013

[jcpp14088-bib-0124] Schmidt‐Kastner, R. , Van Os, J. , Esquivel, G. , Steinbusch, H. , & Rutten, B. (2012). An environmental analysis of genes associated with schizophrenia: Hypoxia and vascular factors as interacting elements in the neurodevelopmental model. Molecular Psychiatry, 17, 1194–1205. 22290124 10.1038/mp.2011.183

[jcpp14088-bib-0125] Schneider, C. , Corrigall, R. , Hayes, D. , Kyriakopoulos, M. , & Frangou, S. (2014). Systematic review of the efficacy and tolerability of clozapine in the treatment of youth with early onset schizophrenia. European Psychiatry, 29, 1–10. 24119631 10.1016/j.eurpsy.2013.08.001

[jcpp14088-bib-0126] Schneider, C. , Papachristou, E. , Wimberley, T. , Gasse, C. , Dima, D. , MacCabe, J.H. , … & Frangou, S. (2015). Clozapine use in childhood and adolescent schizophrenia: A nationwide population‐based study. European Neuropsychopharmacology, 25, 857–863. 25769917 10.1016/j.euroneuro.2015.02.003

[jcpp14088-bib-0127] Schutte, M.J. , Linszen, M.M. , Marschall, T.M. , Koops, S. , van Dellen, E. , Heringa, S.M. , … & Lemstra, A.W. (2020). Hallucinations and other psychotic experiences across diagnoses: A comparison of phenomenological features. Psychiatry Research, 292, 113314. 32731082 10.1016/j.psychres.2020.113314

[jcpp14088-bib-0128] Scott, J. , Chant, D. , Andrews, G. , & McGrath, J. (2006). Psychotic‐like experiences in the general community: The correlates of CIDI psychosis screen items in an Australian sample. Psychological Medicine, 36, 231–238. 16303059 10.1017/S0033291705006392

[jcpp14088-bib-0129] Scott, J. , Martin, G. , Bor, W. , Sawyer, M. , Clark, J. , & McGrath, J. (2009). The prevalence and correlates of hallucinations in Australian adolescents: Results from a national survey. Schizophrenia Research, 107, 179–185. 19046858 10.1016/j.schres.2008.11.002

[jcpp14088-bib-0130] Scott, J.G. , McKeon, G. , Malacova, E. , Curtis, J. , Burgher, B. , Macmillan, I. , … & Parker, S.D. (2022). Quality prescribing in early psychosis: Key pharmacotherapy principles. Australasian Psychiatry, 30, 341–345. 34748711 10.1177/10398562211054656

[jcpp14088-bib-0131] Scott, J.G. , Ross, C.A. , Dorahy, M.J. , Read, J. , & Schäfer, I. (2018). Childhood trauma in psychotic and dissociative disorders. Psychosis, Trauma and Dissociation: Evolving Perspectives on Severe Psychopathology, 141–157. https://onlinelibrary.wiley.com/doi/chapter‐epub/10.1002/9781118585948.ch9.

[jcpp14088-bib-0132] Shah, J.L. , Tandon, N. , & Keshavan, M.S. (2013). Psychosis prediction and clinical utility in familial high‐risk studies: Selective review, synthesis, and implications for early detection and intervention. Early Intervention in Psychiatry, 7, 345–360. 23693118 10.1111/eip.12054 PMC5218827

[jcpp14088-bib-0133] Shevlin, M. , Boyda, D. , Houston, J. , & Murphy, J. (2015). Measurement of the psychosis continuum: Modelling the frequency and distress of subclinical psychotic experiences. Psychosis, 7, 108–118.

[jcpp14088-bib-0134] Simon, A.E. , Velthorst, E. , Nieman, D.H. , Linszen, D. , Umbricht, D. , & de Haan, L. (2011). Ultra high‐risk state for psychosis and non‐transition: A systematic review. Schizophrenia Research, 132, 8–17. 21784618 10.1016/j.schres.2011.07.002

[jcpp14088-bib-0135] Solmi, M. , Radua, J. , Olivola, M. , Croce, E. , Soardo, L. , Salazar de Pablo, G. , … & Kim, J.H. (2022). Age at onset of mental disorders worldwide: Large‐scale meta‐analysis of 192 epidemiological studies. Molecular Psychiatry, 27, 281–295. 34079068 10.1038/s41380-021-01161-7 PMC8960395

[jcpp14088-bib-0136] Spada, G. , Molteni, S. , Pistone, C. , Chiappedi, M. , McGuire, P. , Fusar‐Poli, P. , & Balottin, U. (2016). Identifying children and adolescents at ultra high risk of psychosis in Italian neuropsychiatry services: A feasibility study. European Child and Adolescent Psychiatry, 25, 91–106. 25925786 10.1007/s00787-015-0710-8

[jcpp14088-bib-0137] Sporn, A.L. , Vermani, A. , Greenstein, D.K. , Bobb, A.J. , Spencer, E.P. , Clasen, L.S. , … & Lenane, M.C. (2007). Clozapine treatment of childhood‐onset schizophrenia: Evaluation of effectiveness, adverse effects, and long‐term outcome. Journal of the American Academy of Child & Adolescent Psychiatry, 46, 1349–1356. 17885577 10.1097/chi.0b013e31812eed10

[jcpp14088-bib-0138] Stain, H.J. , Bucci, S. , Baker, A.L. , Carr, V. , Emsley, R. , Halpin, S. , … & Crittenden, K. (2016). A randomised controlled trial of cognitive behaviour therapy versus non‐directive reflective listening for young people at ultra high risk of developing psychosis: The detection and evaluation of psychological therapy (DEPTh) trial. Schizophrenia Research, 176, 212–219. 27554197 10.1016/j.schres.2016.08.008

[jcpp14088-bib-0139] Staines, L. , Healy, C. , Corcoran, P. , Keeley, H. , Coughlan, H. , McMahon, E. , … & Wasserman, C. (2023). Investigating the effectiveness of three school based interventions for preventing psychotic experiences over a year period – A secondary data analysis study of a randomized control trial. BMC Public Health, 23, 219. 36726107 10.1186/s12889-023-15107-x PMC9890687

[jcpp14088-bib-0140] Staines, L. , Healy, C. , Murphy, F. , Byrne, J. , Murphy, J. , Kelleher, I. , … & Cannon, M. (2023). Incidence and persistence of psychotic experiences in the general population: Systematic review and meta‐analysis. Schizophrenia Bulletin, 49, 1007–1021. 37402250 10.1093/schbul/sbad056 PMC10318880

[jcpp14088-bib-0141] Trotta, A. , Arseneault, L. , Caspi, A. , Moffitt, T.E. , Danese, A. , Pariante, C. , & Fisher, H.L. (2020). Mental health and functional outcomes in young adulthood of children with psychotic symptoms: A longitudinal cohort study. Schizophrenia Bulletin, 46, 261–271. 31361314 10.1093/schbul/sbz069 PMC7442396

[jcpp14088-bib-0142] Underwood, R. , Peters, E. , & Kumari, V. (2015). Psychobiology of threat appraisal in the context of psychotic experiences: A selective review. European Psychiatry, 30, 817–829. 26443049 10.1016/j.eurpsy.2015.07.001

[jcpp14088-bib-0143] Upthegrove, R. (2022). From co‐morbidity to transdiagnostic potential and novel immunotherapies for psychosis. Psychiatry Research, 317, 114866. 36201895 10.1016/j.psychres.2022.114866

[jcpp14088-bib-0144] Verdoux, H. , Geddes, J.R. , Takei, N. , Lawrie, S.M. , Bovet, P. , Eagles, J.M. , … & O'Callaghan, E. (1997). Obstetric complications and age at onset in schizophrenia: An international collaborative meta‐analysis of individual patient data. American Journal of Psychiatry, 154, 1220–1227. 9286180 10.1176/ajp.154.9.1220

[jcpp14088-bib-0145] Welham, J. , Scott, J. , Williams, G. , Najman, J. , Bor, W. , O'Callaghan, M. , & McGrath, J. (2009). Emotional and behavioural antecedents of young adults who screen positive for non‐affective psychosis: A 21‐year birth cohort study. Psychological Medicine, 39, 625–634. 18606046 10.1017/S0033291708003760

[jcpp14088-bib-0146] Wigman, J. , Van Os, J. , Abidi, L. , Huibers, M. , Roelofs, J. , Arntz, A. , … & Peeters, F. (2014). Subclinical psychotic experiences and bipolar spectrum features in depression: Association with outcome of psychotherapy. Psychological Medicine, 44, 325–336. 23651602 10.1017/S0033291713000871

[jcpp14088-bib-0147] Wigman, J.T. , Devlin, N. , Kelleher, I. , Murtagh, A. , Harley, M. , Kehoe, A. , … & Cannon, M. (2014). Psychotic symptoms, functioning and coping in adolescents with mental illness. BMC Psychiatry, 14, 1–9. 10.1186/1471-244X-14-97 PMC3976538 24690447

[jcpp14088-bib-0148] Wüsten, C. , Schlier, B. , Jaya, E.S. , Fonseca‐Pedrero, E. , Peters, E. , Verdoux, H. , … & Lincoln, T.M. (2018). Psychotic experiences and related distress: A cross‐national comparison and network analysis based on 7141 participants from 13 countries. Schizophrenia Bulletin, 44, 1185–1194. 29982814 10.1093/schbul/sby087 PMC6192474

[jcpp14088-bib-0149] Wykes, T. , Newton, E. , Landau, S. , Rice, C. , Thompson, N. , & Frangou, S. (2007). Cognitive remediation therapy (CRT) for young early onset patients with schizophrenia: An exploratory randomized controlled trial. Schizophrenia Research, 94, 221–230. 17524620 10.1016/j.schres.2007.03.030

[jcpp14088-bib-0150] Xu, L. , Guo, Y. , Cao, Q. , Li, X. , Mei, T. , Ma, Z. , … & Liu, J. (2020). Predictors of outcome in early onset schizophrenia: A 10‐year follow‐up study. BMC Psychiatry, 20, 1–9. 32059664 10.1186/s12888-020-2484-x PMC7023710

[jcpp14088-bib-0151] Yates, K. , Lång, U. , Cederlöf, M. , Boland, F. , Taylor, P. , Cannon, M. , … & Kelleher, I. (2019). Association of psychotic experiences with subsequent risk of suicidal ideation, suicide attempts, and suicide deaths: A systematic review and meta‐analysis of longitudinal population studies. JAMA Psychiatry, 76, 180–189. 30484818 10.1001/jamapsychiatry.2018.3514 PMC6439738

[jcpp14088-bib-0152] Yung, A.R. , Phillips, L.J. , Yuen, H.P. , & McGorry, P.D. (2004). Risk factors for psychosis in an ultra high‐risk group: Psychopathology and clinical features. Schizophrenia Research, 67, 131–142. 14984872 10.1016/S0920-9964(03)00192-0

[jcpp14088-bib-0153] Zachar, P. , First, M.B. , & Kendler, K.S. (2020). The DSM‐5 proposal for attenuated psychosis syndrome: A history. Psychological Medicine, 50, 920–926. 32234093 10.1017/S0033291720000653

[jcpp14088-bib-0154] Zammit, S. , Kounali, D. , Cannon, M. , David, A.S. , Gunnell, D. , Heron, J. , … & Wolke, D. (2013). Psychotic experiences and psychotic disorders at age 18 in relation to psychotic experiences at age 12 in a longitudinal population‐based cohort study. American Journal of Psychiatry, 170, 742–750. 23639948 10.1176/appi.ajp.2013.12060768

